# Causal inference methods to assess safety upper bounds in randomized trials with noncompliance

**DOI:** 10.1177/1740774515572352

**Published:** 2015-03-01

**Authors:** Yiting Wang, Jesse A Berlin, José Pinheiro, Marsha A Wilcox

**Affiliations:** 1 Janssen Research & Development, LLC, Titusville, NJ, USA; 2 Johnson & Johnson, Titusville, NJ, USA; 3 Janssen Research & Development, LLC, Raritan, NJ, USA

**Keywords:** Noncompliance, major adverse cardiovascular events, safety upper bound, causal survival analysis

## Abstract

**Background:**

Premature discontinuation and other forms of noncompliance with treatment assignment can complicate causal inference of treatment effects in randomized trials. The intent-to-treat analysis gives unbiased estimates for causal effects of treatment assignment on outcome, but may understate potential benefit or harm of actual treatment. The corresponding upper confidence limit can also be underestimated.

**Purpose:**

To compare estimates of the hazard ratio and upper bound of the two-sided 95% confidence interval from causal inference methods that account for noncompliance with those from the intent-to-treat analysis.

**Methods:**

We used simulations with parameters chosen to reflect cardiovascular safety trials of diabetes drugs, with a focus on upper bound estimates relative to 1.3, based on regulatory guidelines. A total of 1000 simulations were run under each parameter combination for a hypothetical trial of 10,000 total subjects randomly assigned to active treatment or control at 1:1 ratio. Noncompliance was considered in the form of treatment discontinuation and cross-over at specified proportions, with an assumed true hazard ratio of 0.9, 1, and 1.3, respectively. Various levels of risk associated with being a non-complier (independent of treatment status) were evaluated. Hazard ratio and upper bound estimates from causal survival analysis and intent-to-treat were obtained from each simulation and summarized under each parameter setting.

**Results:**

Causal analysis estimated the true hazard ratio with little bias in almost all settings examined. Intent-to-treat was unbiased only when the true hazard ratio = 1; otherwise it underestimated both benefit and harm. When upper bound estimates from intent-to-treat were ≥1.3, corresponding estimates from causal analysis were also ≥1.3 in almost 100% of the simulations, regardless of the true hazard ratio. When upper bound estimates from intent-to-treat were <1.3 and the true hazard ratio = 1, corresponding upper bound estimates from causal analysis were ≥1.3 in up to 66% of the simulations under some settings.

**Limitations:**

Simulations cannot cover all scenarios for noncompliance in real randomized trials.

**Conclusion:**

Causal survival analysis was superior to intent-to-treat in estimating the true hazard ratio with respect to bias in the presence of noncompliance. However, its large variance should be considered for safety upper bound exclusion especially when the true hazard ratio = 1. Our simulations provided a broad reference for practical considerations of bias–variance trade-off in dealing with noncompliance in cardiovascular safety trials of diabetes drugs. Further research is warranted for the development and application of causal inference methods in the evaluation of safety upper bounds.

## Introduction

Noncompliance with randomly assigned treatment is commonly encountered in randomized clinical trials^1^ and pragmatic trials.^2^ The intent-to-treat (ITT) analysis compares randomization groups as randomized, and gives unbiased estimates for causal effects of treatment assignment on outcome, but ITT ignores compliance information. When compliance is not perfect, the average causal effect of random treatment assignment may differ from that of non-random receipt of treatment. Although generally endorsed as conservative in estimating efficacy (i.e. underestimation of benefits), ITT may also underestimate harm in safety endpoints.^3–5^ Of particular concern is that the upper confidence limit may also be underestimated. In the interest of better understanding and possibly protecting patient safety, the causal effect of actual exposure to treatment on an adverse outcome among the treated or among those who would comply with treatment may be of primary interest. For example, if an ITT analysis estimated an increased risk of cardiovascular event of up to 20% (i.e. upper bound = 1.2 for the risk ratio) for a drug treatment, based on a clinical trial with 40% of patients not taking the assigned treatment, the true risk may well exceed 30% among patients who actually took it. In practice, it would be useful to understand not just “up to 20% increased risk for all patients who are prescribed the drug,” but also “up to 30% increased risk for patients who take the drug as prescribed.”

Causal inference methods have been developed, which can be applied to evaluate potential safety upper bound in randomized trials with noncompliance.^3^ However, practical application of such methods for this purpose is still limited, compared to other alternatives to ITT such as per protocol, as-treated and/or on-treatment analysis,^6^ as well as various modified ITT analyses.^7^ These other alternative analysis methods may be biased due to post-randomization unmeasured confounding or selection bias^8^ in the relationship between noncompliance and the outcomes of interest, that is, factors that affect compliance may also be independently related to outcomes.^9,10^ For example, previous clinical trials have found that in placebo arms, patients with poor adherence, compared with patients with good adherence, had higher risk of cardiovascular events.^11–14^

The cardiovascular safety of new therapies to treat type 2 diabetes provides an interesting context for the evaluation of safety upper bound in clinical trials with noncompliance. Although lowering of HbA1c (the primary efficacy endpoint) reflects a beneficial effect on the immediate clinical consequences of diabetes, and is reasonably expected to reduce the long-term risk of microvascular complications (e.g. retinopathy, nephropathy), concerns about cardiovascular safety remain.^15^ To exclude an unacceptable cardiovascular risk of new type 2 diabetes therapies, the US Food and Drug Administration guidelines require that the upper bound of the two-sided 95% confidence interval for the estimated risk ratio be less than 1.3 for major adverse cardiovascular events (MACE), which should include cardiovascular mortality, myocardial infarction, and stroke.^15^ A number of large clinical trials of diabetes drugs are being conducted mainly for this purpose.^16–19^ Two of these studies^20,21^ published results recently; both reported approximately 20% premature discontinuation during the trials, and both met the required upper bound of <1.3 by ITT analysis. Although some noncompliance in these trials is probably inevitable, it is unknown what impact such noncompliance may have on the cardiovascular safety upper bound evaluation, and whether ITT or various alternative analyses may address noncompliance appropriately. Potential advantages and limitations of these alternative analyses also need to be better understood.

We conducted a simulation study, using cardiovascular safety trials for type 2 diabetes therapies as a motivating example to (1) evaluate point estimates of cardiovascular risk by ITT and causal inference methods and (2) compare ITT and causal analyses with respect to the estimated 95% upper confidence limit (safety upper bound), specifically whether the estimated upper bound is above or below the threshold of 1.3 under various noncompliance conditions.

## Methods

We simulated a cardiovascular safety trial for a hypothetical type 2 diabetes therapy with 10,000 total subjects randomly assigned to either the treatment or control arm in 1:1 ratio. Cox proportional hazards models were used in the ITT analysis of time to first occurrence of MACE, the outcome of interest. The hazard ratio (HR) was the primary measure of treatment effect. Follow-up was administratively censored when a total of 611 MACE had occurred, to reflect the common use of event-driven trials (i.e. trials in which the duration of study follow-up is not fixed in advance but depends on the number of MACE). The number 611 was chosen to give 90% power for excluding an upper bound of 1.3 for the two-sided 95% confidence interval if the underlying true HR = 1, comparing treatment to control. We also explored a fixed follow-up of 3 years for the hypothetical trial.

Because of our interest in potential harm for patients who would comply with their prescribed treatment, we focused on estimating causal treatment effects that would have been observed under perfect compliance. Using terminology from principal stratification,^22^ we considered three types of potential compliance/noncompliance behaviors: (1) compliers would always take the assigned treatment (or control) throughout the trial, and causal effect (benefit or harm) of the treatment is defined for compliers; (2) always-takers would fully comply with the treatment when assigned to it, but would cross-over to the treatment if assigned to control; (3) never-takers would stop the assigned treatment prematurely before the end of trial, but would fully comply when assigned to control. We did not consider defiers, who would take the active treatment if assigned to control, but would not take the active treatment if assigned to it. For lack of a better term, we used “never-takers” to include not only subjects who would truly never take the assigned treatment (no exposure at all), but also those who may take the assigned treatment initially but would not adhere to it throughout the trial (partial compliance). That is, cross-over (from control to active treatment) noncompliance is captured by “always-takers”, while premature discontinuation of the active treatment at the beginning of or during the trial is captured by “never-takers”.

In practice, the control regimen to be compared with the active treatment of interest may be placebo or standard therapies. In this study, we assumed that the control regimen *per se* had no effects on MACE, although never-takers themselves may have a different risk of MACE compared with compliers. This assumption essentially provided a single reference level against which the risk of MACE for the active treatment is compared, consistent with the practice where one new type 2 diabetes drug is compared with alternative standard therapies as control. For example, if the control regimen is placebo on the background of metformin plus glimepiride, it may be assumed that the risk of MACE remains largely unchanged (from some baseline level that may be different from that of compliers) for a patient who discontinues glimepiride, or who takes glipizide instead. By randomization, the distribution of trial participants with each type of potential compliance was expected to balance across the treatment and control groups. However, the compliance type of all individuals is not identifiable in a particular trial. For example, an always-taker assigned to the treatment arm cannot be distinguished from a complier as both would exhibit perfect compliance in that trial. Likewise, a never-taker assigned to the control arm cannot be distinguished from a complier assigned to control. To cover a broad range of noncompliance reported in clinical trials of type 2 diabetes drugs (see Appendix 2, available online),^23–26^ the following triples were used to specify the proportions of compliers, never-takers, and always-takers, respectively: 〈80%, 20%, 0%〉, 〈60%, 40%, 0%〉, 〈80%, 10%, 10%〉, and 〈60%, 20%, 20%〉 (the proportions must sum to 1 because we assumed that all trial participants fall into one of these three categories). By randomization, each set of these triples was equally applied across the treatment and control groups.

We considered both all-or-none^27^ and partial compliance. In all-or-none compliance, a never-taker assigned to treatment would fail to take the treatment at the very beginning of the trial (time zero), whereas an always-taker assigned to control would initiate treatment at time zero. In partial compliance, a never-taker may initially take the assigned treatment but would discontinue prematurely (before the occurrence of MACE or end of trial). Likewise, an always-taker assigned to the control arm may cross-over anytime during the follow-up and remain on treatment for the rest of the trial. In partial noncompliance, the timing of treatment discontinuation or cross-over was assumed to be uniformly distributed over potential (or counterfactual) time to MACE. Potential time to MACE was assumed to follow a Weibull distribution with survival function exp(–λt^α^), where λ is the scale parameter and α is the shape parameter (see Appendix 1). The Weibull distribution was chosen because it satisfies both the proportional hazards assumption and the accelerated failure time assumption. The Weibull parameters were chosen to reflect an initial annual cardiovascular risk of about 2% among type 2 diabetes, which rose during follow-up with increasing age and duration of diabetes.^28,29^ We chose a common α = 1.22 for all subjects, λ_0_ = 0.024 for untreated compliers, and λ_1_ = λ_0_*HR for treated compliers, where HR was the true causal HR comparing treatment with control and took on values of 0.9, 1, or 1.3 (see Appendix 3a-3c (available online) for supplemental analyses when HR = 1.1 or 1.2). We let the Weibull scale parameters for the untreated never-takers and untreated always-takers be λ_0_*RR_n_ and λ_0_*RR_a_, respectively, where RR_n_ and RR_a_ denote the risk ratio for MACE of never-takers and always-takers, respectively, compared with compliers in the absence of treatment. Previous clinical trials have reported about 50% lower risk of cardiovascular events associated with good versus poor placebo adherence;^11–14^ therefore, we chose RR_n_ = 1 or 2 and RR_a_ = 1 or 2.

For simplicity, we assumed no latency or carry-over of treatment effects so that a change in treatment (discontinuation or cross-over) led to an immediate change in hazard/survival probability that corresponded to the “current” exposure to treatment or control.

In ITT analyses, we fit both Cox proportional hazards and Weibull survival models to estimate the effects of treatment using an indicator variable for initial random assignment of treatment or control. The estimates for the shape parameter α from the Weibull survival models were also saved for use in subsequent analyses (i.e. converting time ratio (TR) to HR estimates) during the simulations.

In causal survival analysis, we employed two methods described heuristically below (see Kim and White,^30^ Loeys and Goetghebeur,^31^ Robins and Tsiatis^32^ and White et al.^33^ for technical details).

The first method estimated Compliers PROPortional Hazards Effect of Treatment (“C-PROPHET”).^30,31^ Briefly, this method assumes all-or-none compliance, with only compliers and never-takers, but no always-takers (as would be the case in most pre-approval drug trials when the control group has no access to the treatment), and no effect on outcome by randomization *per se*. The causal HR of the treatment for compliers cannot be directly estimated because compliers cannot all be identified (explained above). However, because never-takers assigned to the treatment arm can be identified, their proportion and Kaplan–Meier survival function can both be estimated. Likewise, the Kaplan–Meier survival function for treated compliers can be estimated from the treatment arm. The survival function for untreated compliers can then be derived under an assumed HR. Given all these, the expected number of events on the control arm can be predicted using the mixture of survival functions of never-takers and untreated compliers. The solution for the causal HR is found iteratively by matching the predicted with the observed number of events on the control arm.

The second method estimated the causal survival TR under a structural nested accelerated failure time (SNAFT) model.^32,33^ Suppose the observed event time for a patient was t = t_0_ + t_1_, where t_0_ and t_1_ were the lengths of time that the patient spent off and on treatment before the event, respectively. The parameter TR relates t with a potential or counterfactual event time u = t_0_ + t_1_/TR that would have been observed in the absence of treatment and represents a factor by which time to MACE is changed by treatment. By randomization, TR is estimated iteratively as the value at which the counterfactual failure time without treatment is balanced between the treatment and control groups according to the log-rank test. For Weibull distributions, HR = (1/TR)^α^ so that the causal HR can be estimated by plugging in the TR and α (from ITT analysis) estimates.

Under all-or-none compliance, both methods are applicable and both are consistent. We only present results from C-PROPHET under this scenario because the HR was directly estimated (not converted from TR) and because variance from C-PROPHET was uniformly smaller than (although similar in magnitude to) that from the SNAFT method in our simulations and in previous studies.^31^ For partial compliance or treatment cross-over, only the SNAFT method was applicable and applied.

We ran 1000 simulations (hypothetical trial data generation and analysis) under each combination of the parameters and summarized results across these 1000 sets of analyses. All simulations were conducted in STATA^®^, v12.1, College Station, Texas. HR point estimates and confidence limits from ITT and causal survival analyses were summarized on the natural-log (denoted “ln”) scale by mean, standard deviation, median, 25th and 75th percentiles, as well as minimum and maximum. Bias in point estimates was defined as the difference between the mean of ln(estimated HR) and ln(true HR). For simplicity and for practical purposes, bias of small magnitude (absolute value ≤0.009) from the simulations was considered as negligible. For example, when the true HR = 1, any estimated HR between 0.991 and 1.009 would meet this condition with a relative bias of <1% on the original HR scale. Simplified box-plots showing key statistics (median, 25th and 75th percentiles, 1.5 times above and below interquartile range, as well as the minimum and maximum) of the HR point estimates are presented on the original scale. HR upper bound estimates from each simulation were also recorded as ≥1.3 or <1.3. The percentage of times out of 1000 simulations that the upper bound estimates from ITT and causal survival analysis were in agreement (both ≥1.3 or both <1.3) or disagreement (one ≥1.3 whereas the other <1.3 or vice versa) was plotted using bar charts.

Although our simulation parameters (including sample size) were chosen to reflect situations in cardiovascular safety trials of type 2 diabetes treatment,^16–19^ additional parameter settings were explored (including different sample sizes, various allocation ratios for treatment and control, different proportions of compliers, never-takers, and always-takers, different seeds for random number generation in STATA^®^, and different HR and Weibull parameters) on a post hoc basis to assess the generalizability of our study findings. Since it is impractical to cover all interesting scenarios in one simulation study,^34^ we discuss some additional situations not represented in our simulations later in the article.

See Appendix 4 (available online) for STATA^®^ programs and supplemental results.

## Results

### Point estimates

**Figure 1.**
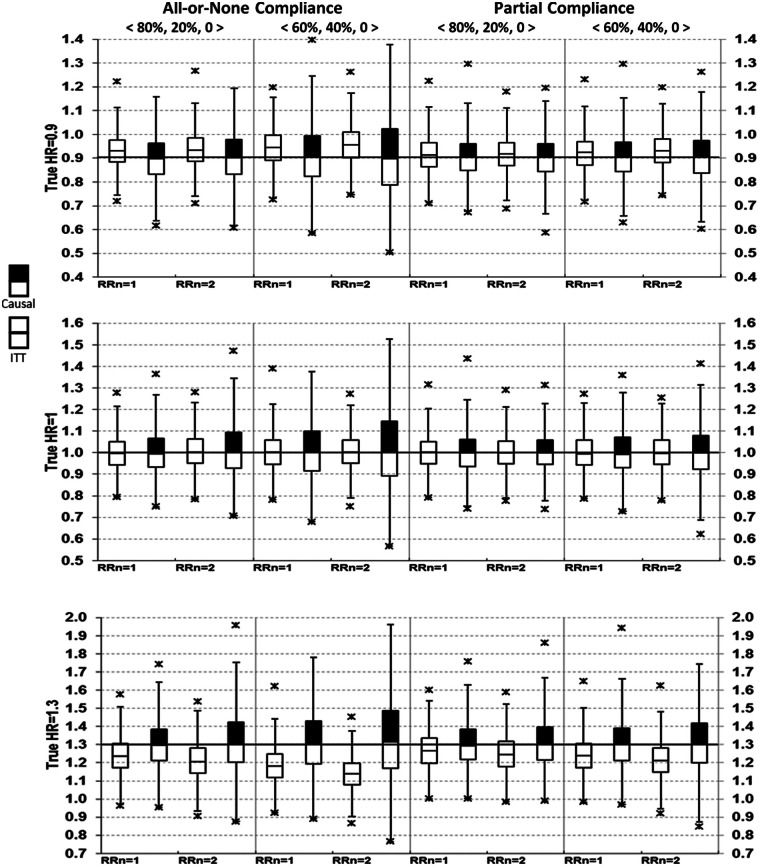
Comparing intent-to-treat (ITT) with causal survival analysis in hazard ratio (HR) point estimates (no always-takers). Simplified box-plots show the median, 25th and 75th percentiles, 1.5 times above and below interquartile range of the HR point estimates. Limited by the graphical scale and space, the minimum and maximum (shown by stars) of HR estimates are not displayed for all scenarios, but all numerical results are provided in the Appendix 3a-3c (available online). Causal analysis (shown with top-filled boxes) was based on the method of Loeys (2003). Compliance proportions: 〈80%, 20%, 0%〉 for 80% compliers and 20% never-takers; 〈60%, 40%, 0%〉 for 60% compliers and 40% never-takers. RR_n_ denotes risk ratio for major adverse cardiovascular events (MACE) comparing untreated never-takers to untreated compliers.

**Figure 2.**
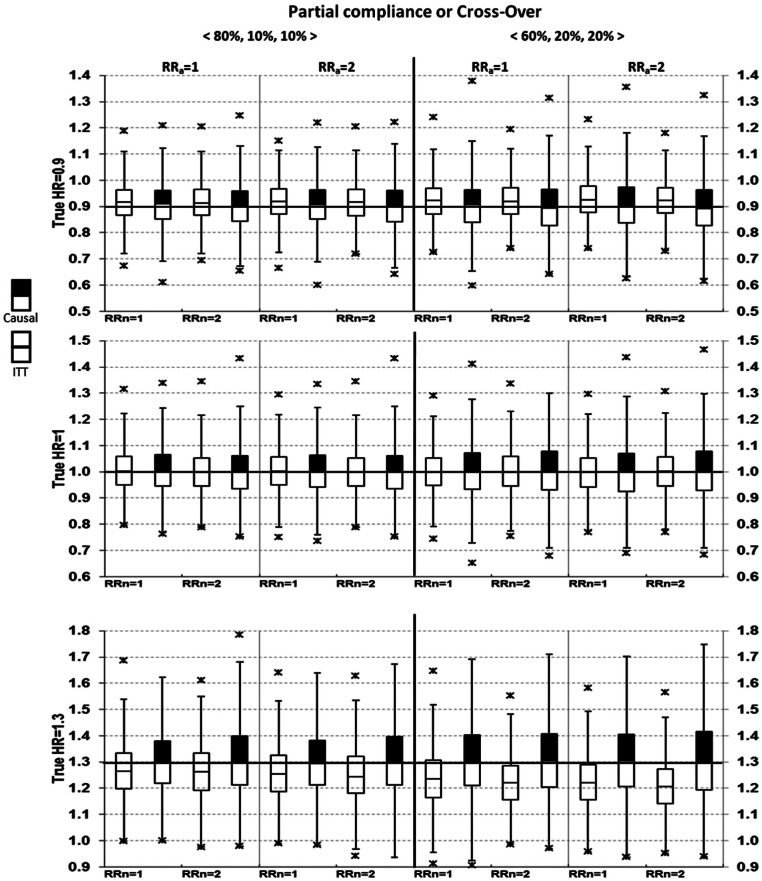
Comparing intent-to-treat (ITT) with causal survival analysis in hazard ratio (HR) point estimates (with always-takers). Simplified box-plots show the median, 25th and 75th percentiles, 1.5 times above and below interquartile range of the HR point estimates. Limited by the graphical scale and space, the minimum and maximum (shown by stars) of HR point estimates are not displayed for all scenarios, but all numerical results are provided in the Appendix 3a-3c (available online). Causal analysis (shown with top-filled boxes) was based on the method of Robins (1991). Compliance proportions: 〈80%, 10%, 10%〉 for 80% compliers, 10% never-takers, and 10% always-takers; 〈60%, 20%, 20%〉 for 60% compliers, 20% never-takers, and 20% always-takers. RR_n_ denotes risk ratio for major adverse cardiovascular events (MACE) comparing untreated never-takers and untreated compliers; RR_a_ denotes risk ratio for MACE comparing untreated always-takers with untreated compliers.

Box-plots for HR point estimates are shown in Figures 1 and 2.

Results from the ITT analyses were as expected. Bias = 0 when the true HR = 1 (i.e. treatment had no effects on MACE). Bias <0 when the true HR >1, and bias >0 when the true HR <1, therefore ITT underestimated both harm and benefits. This is seen as the medians shifted toward the null value of 1. Note that although only medians were displayed in the box-plots, the corresponding mean estimates were virtually identical to the medians in all simulations (data not shown).

From causal survival analysis, bias = 0 in all pre-specified scenarios except for one, which assumed the true HR = 1.3, all-or-none compliance with 40% of subjects being never-takers, who had a risk ratio = 2 for MACE compared with untreated compliers (Figure 1, lower left). Using various random seeds under this parameter setting, we consistently observed bias of about 0.02, with an estimated HR between 1.32 and 1.33. In addition to post hoc explorations, bias from causal survival analysis was also slightly >1% of the true HR for all-or-none compliance under more extreme conditions, such as when never-takers had a risk ratio of ≥2.5 for MACE compared with untreated compliers or when the proportion of never-takers was ≥60% (results not shown).

## Upper bound

**Figure 3.**
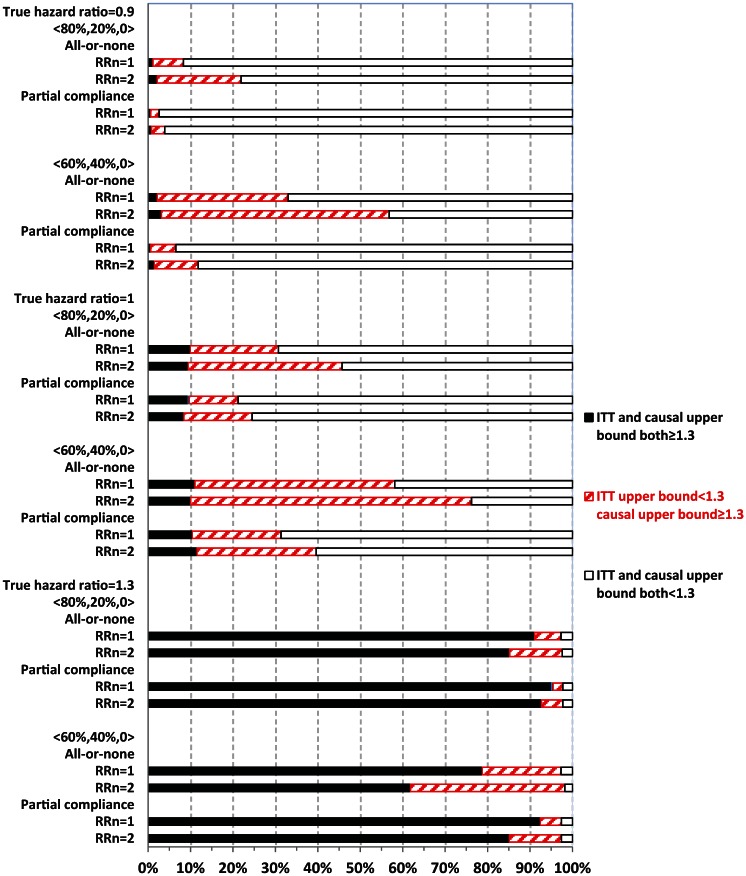
Comparing intent-to-treat (ITT) with causal survival analysis in safety upper bound estimates relative to 1.3 (no always-takers). The vertical axis displays 24 different combinations of the simulation parameters. Compliance proportions were represented by 〈80%, 20%, 0%〉 for 80% compliers and 20% never-takers; 〈60%, 40%, 0%〉 for 60% compliers and 40% never-takers. RR_n_ denotes risk ratio for major adverse cardiovascular events (MACE) comparing untreated never-takers to untreated compliers. Each of the 24 horizontal bars shows the respective percentages (read from horizontal axis) of the 1000 simulations under each corresponding parameter setting: with both ITT and causal upper bound of ≥1.3 (represented by the solid black portion on the horizontal bar) or with ITT upper bound of <1.3 but causal upper bound of ≥1.3 (represented by the portion marked by red forward slashes) or with both ITT and causal upper bound of <1.3 (represented by the empty portion on the horizontal bar).

**Figure 4.**
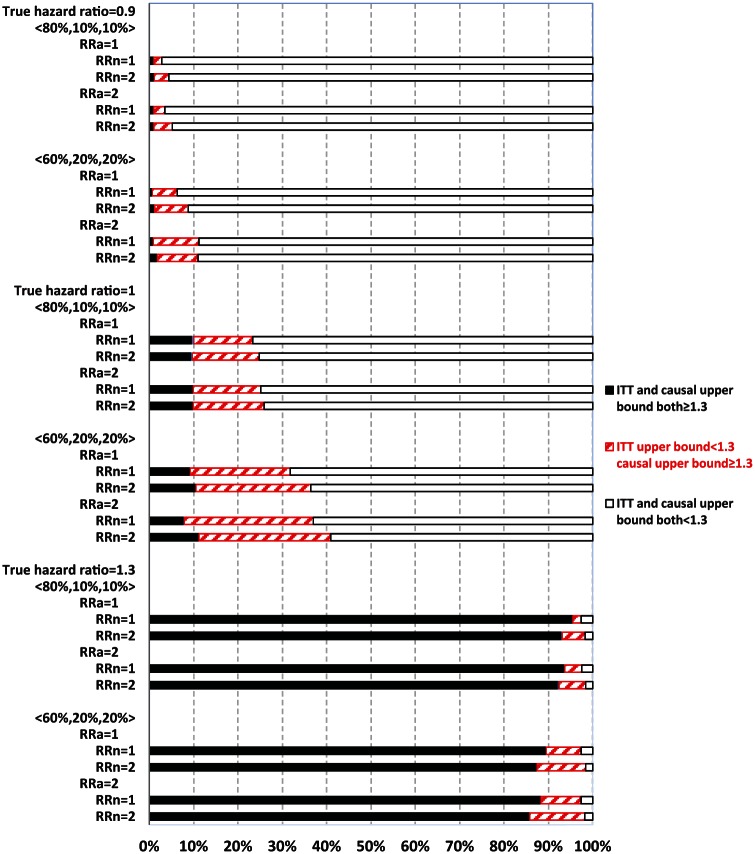
Comparing intent-to-treat (ITT) with causal survival analysis in safety upper bound estimates relative to 1.3 (with always-takers). The vertical axis displays 24 different combinations of the simulation parameters. Compliance proportions were represented by 〈80%, 10%, 10%〉 for 80% compliers, 10% never-takers, and 10% always-takers; 〈60%, 20%, 20%〉 for 60% compliers, 20% never-takers, and 20% always-takers. RR_a_ denotes the risk ratio for major adverse cardiovascular events (MACE) comparing untreated always-takers to untreated compliers, and RR_n_ denotes the risk ratio for MACE comparing untreated never-takers to untreated compliers. Each of the 24 horizontal bars shows the respective percentages (read from horizontal axis) of the 1000 simulations under each corresponding parameter setting: with both ITT and causal upper bound of ≥1.3 (represented by the solid black portion on the horizontal bar), or with ITT upper bound of <1.3 but causal upper bound of ≥1.3 (represented by the portion marked by red forward slashes), or with both ITT and causal upper bound of <1.3 (represented by the empty portion on the horizontal bar).

Figures 3 and 4 display the percentage of times out of 1000 simulations when the upper bound estimates from ITT and causal analysis were in agreement (both ≥1.3 or both <1.3) or disagreement (<1.3 from ITT, whereas ≥1.3 from causal analysis). There was practically no disagreement (0%) from causal analysis when ITT upper bound was ≥1.3.

When ITT upper bound is <1.3, the percentage of times causal upper bound is ≥1.3 depended upon the true HR and other parameters. Causal analysis disagreed with ITT upper bound of <1.3 more often when never-takers had greater baseline risk of MACE than compliers (RR_n_ = 2) than otherwise (RR_n_ = 1), but less often in partial compliance compared with all-or-none compliance. For example, with a true HR = 1.3 and 80% compliers, causal analysis disagreed with ITT upper bound of <1.3 in 12.5% and 6.2% of the 1000 simulations corresponding to RR_n_ = 2 and 1, respectively.

When the true HR = 1.3, the overall probability of ITT upper bound of ≥1.3 was high (>80%) in most scenarios, except for when it was 62% under all-or-none compliance with 40% never-takers, who had twofold baseline risk of MACE compared with compliers (RR_n_ = 2, Figure 3). Both ITT and causal upper bound were <1.3 in roughly 3% of simulations. Therefore, when treatment truly elevated risk of MACE above the threshold of 1.3, as set forth by regulatory guidelines, ITT could show this by estimating upper bound as ≥1.3 with a high probability. Causal analysis shows this above and beyond ITT (when ITT upper bound is <1.3) by up to an additional 12% of the time (RR_n_ = 2), so that the chance of passing the regulatory hurdle, despite having a true HR of 1.3, was roughly 3%. In contrast, when the true HR = 1, ITT and causal upper bound were both ≥1.3 in roughly 10% of the 1000 simulations, while causal upper bound was ≥1.3 and ITT upper bound was <1.3 for 36% (RR_n_ = 2) and 21% (RR_n_ = 1) of the times. Therefore, even when the true HR = 1, causal analysis alone could suggest an unacceptable risk of MACE for the treatment 46% of the time (RR_n_ = 2). When the true HR = 0.9, ITT upper bound is ≥1.3 in less than 2% of the simulations, while causal upper bound is ≥1.3 and ITT upper bound is <1.3 in 20% (RR_n_ = 2) and 7% (RR_n_ = 1) of simulations. Therefore, when treatment actually lowered the risk of MACE, the chance of causal or ITT upper bound of ≥1.3 was not excessively high (although 20% is non-trivial).

## Discussion

We evaluated the potential impact of noncompliance in randomized trials on safety upper bound evaluation, using simulations to reflect common scenarios in cardiovascular safety studies for type 2 diabetes drug development. For sufficiently powered studies, the probability of correctly excluding upper bound of 1.3 by ITT analysis was good when the true HR = 1.3 in most of these scenarios. Although causal survival analysis consistently estimated the true HR, the variance was large and the probability of suggesting upper bound of ≥1.3 was high, especially when the true HR = 1.

Although causal survival analysis improved safety upper bound exclusion above ITT when the true HR = 1.3, it could lead to “over-correction” with a large probability of causal upper bound of ≥1.3 when the true HR = 1. This mainly resulted from a large variance of the causal analysis methods. Part of this large variance may be explained by the reduced sample size of compliers for inferring complier-treatment effects, but part of it may be inherent in the methods themselves. The relative contribution of these sources is unknown, and we are not aware of an established minimum variance that can be achieved for the causal estimators in general, except under particular settings.^35^ Although the variance of causal upper bound estimates was similar when the true HR = 1 to that when the true HR = 1.3 (other parameters equal), the practical impact on the percentage of times causal upper bound is ≥1.3 when the true HR = 1 is a legitimate concern. A “ceiling” effect might explain this different impact, with more “room” (i.e. percentage) left for causal estimates to disagree with ITT when the true HR = 1 than when the true HR = 1.3. Although a relative large variance, by itself, would not preclude application of a method, the usual trade-off between variance and bias needs careful consideration. Further research is warranted to try to reduce the variance associated with bias correction.

Our observation of the large variance in the causal inference estimates is consistent with previous studies. For example, instrumental variable (IV) estimates were shown to have a larger variance compared with per protocol or as-treated analyses.^36^ Although we did not consider covariate adjustment (not yet implemented in the C-PROPHET or SNAFT STATA^®^ modules), it can reduce bias and improve precision for both causal and alternative methods.^36^

We took the principal stratification approach^22^ to noncompliance in clinical trials because our focus was potential harmful effects on compliers. However, this approach has its limitations, for example, the compliers are generally not distinguishable from always-/never-takers in observed data.^37,38^ Some simplifying assumptions may also be needed for its application. For example, we assumed that standard type 2 diabetes therapies had no effects on MACE (at least for the trial duration) so that the active treatment can be compared with a common reference level. This assumption may hold approximately because standard type 2 diabetes therapies have not demonstrated significant cardio-protective effects.^39^ If one is convinced of any differential effects among these standard therapies on the risk of MACE, additional principal strata may be necessary to account for never-takers who take these different standard therapies or none at all. Although the assumption of no defiers appears to be suitable for most diabetes trials, Bayesian inference methods^40^ may be applied in situations when there are defiers.

Other causal inference methods to address trial noncompliance may include IV survival analysis,^41^ iterative parameter estimation algorithm,^42^ and parametric randomization-based methods.^37^ Some of these methods have been compared with the two methods (C-PROPHET and SNAFT) employed here by simulations for addressing treatment switching^43^ and time-varying noncompliance^38^ in randomized trials. The choice of a particular method should take into consideration the type of noncompliance, distributional assumptions, and bias–variance trade-off, as well as other relevant issues. If the overall average causal treatment effect is of interest, linear programming methods can be used to derive nonparametric bounds in clinical trials with noncompliance.^44^ We employed the two methods based on their relatively simple assumptions (both taking advantage of baseline randomization to address noncompliance) and ease of implementation. If one is able to justify the (strong) assumption of no unmeasured confounding during study follow-up (essentially sequential randomization at baseline and beyond, such as with adequate data collection and covariate adjustment), marginal structural models may also be applied.^5^

Our choice of HR as the outcome measure of interest and simulation parameters were guided by cardiovascular safety studies for diabetes drugs.^23–25^ We note that formal dedicated cardiovascular safety trials for some of the type 2 diabetes drugs are still ongoing,^17–19^ and final results are not yet available. We simulated a single large trial of 10,000 subjects (reflecting the overall large sample size required); however, we note that a meta-analysis of multiple controlled trials is acceptable by regulatory guidelines,^15^ and may be necessary in practice. One approach to safety evaluation that accounts for different noncompliance across multiple trials is to apply causal inference methods on individual studies first and then summarize over the compliance-adjusted HR. Bayesian methods for meta-analysis using genetic IVs^21^ may be useful if they can be adapted for survival analysis.

While we only simulated Weibull survival distributions, the C-PROPHET^31^ method performed well (in terms of bias and coverage) under survival distributions that satisfied either proportional hazards (piecewise exponential) or accelerated-failure-time (log-normal) assumptions or both (exponential distribution). The SNAFT model is semi-parametric and can model various distributions including log-normal, log-logistic, inverse Gaussian, and gamma. We did not consider these alternative distributions, but note that underlying assumptions for any particular distribution should be checked and alternatives sought if violations of the assumptions (e.g. proportional hazards) are of concern. We did not consider additional complexities in causal survival analysis, such as treatment-by-covariate interaction,^45^ switching to non-trial treatment,^46^ non-administrative censoring and other reasons for discontinuation (e.g. rescue therapy, intolerance or adverse events), more than two dosing and/or treatment arms (including active comparators), and missing data. Therefore, further research is needed to guide the practical application of various methods and techniques for safety upper bound evaluation.

## Supplementary Material

Supplementary material

## References

[1] Dodd S White IR Williamson P Nonadherence to treatment protocol in published randomised controlled trials: a review. Trials 2012; 13: 84. 22709676 10.1186/1745-6215-13-84 PMC3492022

[2] Reynolds RF Lem JA Gatto NM Is the large simple trial design used for comparative, post-approval safety research? A review of a clinical trials registry and the published literature. Drug Saf 2011; 34: 799–820. 21879776 10.2165/11593820-000000000-00000

[3] Greenland S Lanes S Jara M Estimating effects from randomized trials with discontinuations: the need for intent-to-treat design and G-estimation. Clin Trials 2008; 5: 5–13. 18283074 10.1177/1740774507087703

[4] Toh S Hernan MA Causal inference from longitudinal studies with baseline randomization. Int J Biostat 2008; 4: Article 22. 10.2202/1557-4679.1117 PMC2835458 20231914

[5] Toh S Hernandez-Diaz S Logan R Estimating absolute risks in the presence of nonadherence: an application to a follow-up study with baseline randomization. Epidemiology 2010; 21: 528–539. 20526200 10.1097/EDE.0b013e3181df1b69 PMC3315056

[6] Hernan MA Hernandez-Diaz S Beyond the intention-to-treat in comparative effectiveness research. Clin Trials 2012; 9: 48–55. 21948059 10.1177/1740774511420743 PMC3731071

[7] Montedori A Bonacini MI Casazza G Modified versus standard intention-to-treat reporting: are there differences in methodological quality, sponsorship, and findings in randomized trials? A cross-sectional study. Trials 2011; 12: 58. 21356072 10.1186/1745-6215-12-58 PMC3055831

[8] Rosenbaum PR The consequences of adjustment for a concomitant variable that has been affected by the treatment. J R Stat Soc A Stat Soc 1984; 147: 656–666.

[9] Sheiner LB Rubin DB Intention-to-treat analysis and the goals of clinical trials. Clin Pharmacol Ther 1995; 57: 6–15. 7828382 10.1016/0009-9236(95)90260-0

[10] Frangakis CE Rubin DB Addressing complications of intention-to-treat analysis in the combined presence of all-or-none treatment-noncompliance and subsequent missing outcomes. Biometrika 1999; 86: 365–379.

[11] Avins AL Pressman A Ackerson L Placebo adherence and its association with morbidity and mortality in the studies of left ventricular dysfunction. J Gen Intern Med 2010; 25: 1275–1281. 20706875 10.1007/s11606-010-1477-8 PMC2988150

[12] Curtis JR Larson JC Delzell E Placebo adherence, clinical outcomes, and mortality in the women’s health initiative randomized hormone therapy trials. Med Care 2011; 49: 427–435. 21422960 10.1097/MLR.0b013e318207ed9e PMC4217207

[13] Glynn RJ Buring JE Manson JE Adherence to aspirin in the prevention of myocardial infarction. The Physicians’ Health Study. Arch Intern Med 1994; 154: 2649–2657. 7993148 10.1001/archinte.1994.00420230032005

[14] Granger BB Swedberg K Ekman I Adherence to candesartan and placebo and outcomes in chronic heart failure in the CHARM programme: double-blind, randomised, controlled clinical trial. Lancet 2005; 366: 2005–2011. 16338449 10.1016/S0140-6736(05)67760-4

[15] Administration UDoHaHSFaD. Guidance for industry: diabetes mellitus—evaluating cardiovascular risk in new antidiabetic therapies to treat type 2 diabetes, 2008 Silver Spring, MD: Center for Drug Evaluation and Research (CDER).

[16] Bailey CJ Interpreting adverse signals in diabetes drug development programs. Diabetes Care 2013; 36: 2098–2106. 23695817 10.2337/dc13-0182 PMC3687324

[17] Fonseca VA Ongoing clinical trials evaluating the cardiovascular safety and efficacy of therapeutic approaches to diabetes mellitus. Am J Cardiol 2011; 108: 52B–58B. 21802581 10.1016/j.amjcard.2011.03.016

[18] Gore MO McGuire DK Drugs for type 2 diabetes mellitus: the imperative for cardiovascular outcome assessment. Diab Vasc Dis Res 2012; 9: 85–88. 22496441 10.1177/1479164112441527

[19] Hirshberg B Raz I Impact of the U.S. Food and Drug Administration cardiovascular assessment requirements on the development of novel antidiabetes drugs. Diabetes Care 2011; 34(Suppl. 2): S101–S106. 21525438 10.2337/dc11-s202 PMC3632144

[20] Scirica BM Bhatt DL Braunwald E Saxagliptin and cardiovascular outcomes in patients with type 2 diabetes mellitus. N Engl J Med 2013; 369: 1317–1326. 23992601 10.1056/NEJMoa1307684

[21] White WB Cannon CP Heller SR Alogliptin after acute coronary syndrome in patients with type 2 diabetes. N Engl J Med 2013; 369: 1327–1335. 23992602 10.1056/NEJMoa1305889

[22] Frangakis CE Rubin DB Principal stratification in causal inference. Biometrics 2002; 58: 21–29. 11890317 10.1111/j.0006-341x.2002.00021.x PMC4137767

[23] Gaziano JM Cincotta AH Vinik A Effect of bromocriptine-QR (a quick-release formulation of bromocriptine mesylate) on major adverse cardiovascular events in type 2 diabetes subjects. J Am Heart Assoc 2012; 1: e002279. 23316290 10.1161/JAHA.112.002279 PMC3541616

[24] Investigators OT Gerstein HC Bosch J Basal insulin and cardiovascular and other outcomes in dysglycemia. N Engl J Med 2012; 367: 319–328. 22686416 10.1056/NEJMoa1203858

[25] Kendall DM Riddle MC Rosenstock J Effects of exenatide (exendin-4) on glycemic control over 30 weeks in patients with type 2 diabetes treated with metformin and a sulfonylurea. Diabetes Care 2005; 28: 1083–1091. 15855571 10.2337/diacare.28.5.1083

[26] Williams-Herman D Engel SS Round E Safety and tolerability of sitagliptin in clinical studies: a pooled analysis of data from 10,246 patients with type 2 diabetes. BMC Endocr Disord 2010; 10: 7. 20412573 10.1186/1472-6823-10-7 PMC3161395

[27] Baker SG Compliance, all-or-none. In Kotz S Read CR Banks DL (eds) The encyclopedia of statistical science. New York: John Wiley & Sons, 1997, pp. 134–138.

[28] Fox CS Pencina MJ Wilson PW Lifetime risk of cardiovascular disease among individuals with and without diabetes stratified by obesity status in the Framingham heart study. Diabetes Care 2008; 31: 1582–1584. 18458146 10.2337/dc08-0025 PMC2494632

[29] Kengne AP Patel A Marre M Contemporary model for cardiovascular risk prediction in people with type 2 diabetes. Eur J Cardiovasc Prev Rehabil 2011; 18: 393–398. 21450612 10.1177/1741826710394270

[30] Kim LG White IR Compliance-adjusted intervention effects in survival data. Stata J 2004; 4: 257–264.

[31] Loeys T Goetghebeur E A causal proportional hazards estimator for the effect of treatment actually received in a randomized trial with all-or-nothing compliance. Biometrics 2003; 59: 100–105. 12762446 10.1111/1541-0420.00012

[32] Robins JM Tsiatis AA Correcting for non-compliance in randomized trials using rank preserving structural failure time models. Comm Stat Theory Methods 1991; 20: 2609–2631.

[33] White IR Walker S Babiker A Strbee: randomization-based efficacy estimator. Stata J 2002; 2: 140–150.

[34] Maldonado G Greenland S The importance of critically interpreting simulation studies. Epidemiology 1997; 8: 453–456. 9209864

[35] Robins J Tsiatis A Semiparametric estimation of an accelerated failure time model with time-dependent covariates. Biometrika 1992; 79: 311–319.

[36] Little RJ Long Q Lin X A comparison of methods for estimating the causal effect of a treatment in randomized clinical trials subject to noncompliance. Biometrics 2009; 65: 640–649. 18510650 10.1111/j.1541-0420.2008.01066.x

[37] Pearl J Principal stratification—a goal or a tool? Int J Biostat 2011; 7: 20. 21556288 10.2202/1557-4679.1322 PMC3083139

[38] Vanderweele TJ Principal stratification—uses and limitations. Int J Biostat 2011; 7: pii: Article 28. 10.2202/1557-4679.1329 PMC3154088 21841939

[39] Skyler JS Bergenstal R Bonow RO Intensive glycemic control and the prevention of cardiovascular events: implications of the ACCORD, ADVANCE, and VA diabetes trials: a position statement of the American Diabetes Association and a scientific statement of the American College of Cardiology Foundation and the American Heart Association. Circulation 2009; 119: 351–357. 19095622 10.1161/CIRCULATIONAHA.108.191305

[40] Imbens GW Rubin DB Bayesian inference for causal effects in randomized experiments with noncompliance. Ann Stat 1997; 25: 305–327.

[41] Stukel TA Fisher ES Wennberg DE Analysis of observational studies in the presence of treatment selection bias: effects of invasive cardiac management on AMI survival using propensity score and instrumental variable methods. JAMA 2007; 297: 278–285. 17227979 10.1001/jama.297.3.278 PMC2170524

[42] Gilbert PB Hudgens MG Wolfson J Commentary on “Principal stratification—a goal or a tool?” by Judea Pearl. Int J Biostat 2011; 7: Article 36. 10.2202/1557-4679.1341 PMC3204668 22049267

[43] Shrier I Kaufman JS Platt RW Principal stratification: a broader vision. Int J Biostat 2013; 9: 307–313. 24402884 10.1515/ijb-2013-0045

[44] Balke A Pearl J Bounds on treatment effects from studies with imperfect compliance. J Am Stat Assoc 1997; 92: 1172–1176.

[45] Matsuyama Y A comparison of the results of intent-to-treat, per-protocol, and g-estimation in the presence of non-random treatment changes in a time-to-event non-inferiority trial. Stat Med 2010; 29: 2107–2116. 20552682 10.1002/sim.3987

[46] Bond SJ White IR Estimating causal effects using prior information on nontrial treatments. Clin Trials 2010; 7: 664–676. 20817650 10.1177/1740774510382439 PMC3131117

