# Supplementary material for: Causal inference methods to assess safety upper bounds in randomized trials with noncompliance
Source: Clin Trials. 2015 Mar 1;12(3):265–75. doi: 10.1177/1740774515572352 (PMC4420771; doi:10.1177/1740774515572352)
Supplement: Supplementary material [file CTJ_572352_supplemental_data.pdf]

Table of Contents

Appendix 1. Explicit expressions for the survival functions by compliance class (principal strata) 1

Appendix 2. Noncompliance rate referenced for simulation parameters[‡] 2

Appendix 3a. Results under all-or-none compliance, summarized across 1,000 simulations under each setting (indexed by Set) 5

Appendix 3b. Results under partial noncompliance, with no always-takers, summarized across 1,000 simulations under each setting (indexed by Set) 16

Appendix 3c. Results when there are always-takers, summarized across 1,000 simulations under each setting (indexed by set) 23

Appendix 4. STATA® programming codes........ 50

Preparations:........ 50

Stata Codes for running simulations, 3 separate modules 50

Module 1: All-or-none noncompliance 50

Module 2: Partial compliance, no always-takers 61

Module 3: Cross-over(always-taker), premature discontinuation (never-taker) 71

Appendix 1. Explicit expressions for the survival functions by compliance class (principal strata)

Compliance class (Principal Strata)	Treatment exposure	Survival function, S(t)=Prob (T>t given compliance class and treatment exposure)	Some examples[‡] for the cumulative risk of MACE (i.e. 1-S(t)) at the end of year		
			Year 1	Year 3	Year 5
Complier	1	$\exp(-HR*\lambda_0*t^{\alpha})$	3.1%	11.2%	19.9%
	0	$\exp(-\lambda_0*t^{\alpha})$	2.4%	8.8%	15.7%
Never-taker	1 or 0	$\exp(-RR_n*\lambda_0*t^{\alpha})$	4.7%	16.8%	29.0%
Always-taker	1 or 0	$\exp(-RR_a*HR*\lambda_0*t^{\alpha})$	6.0%	21.2%	35.9%

‡We chose a common α=1.22 for all subjects, λ_0=0.024 for untreated compliers, and λ_1= λ_0*HR for treated compliers, where HR was the true causal hazard ratio comparing treatment with control, in the examples we assumed HR=1.3. RR_n and RR_a denote the risk ratio for major cardiovascular events (MACE) of never-takers and always-takers, respectively, compared with compliers in the absence of treatment. In the examples, we assumed RR_n=2, RR_a=2.

Appendix 2. Noncompliance rate referenced for simulation parameters‡

Active Treatment	% Premature discontinuation (averaged across multiple trials not dedicated for cardiovascular safety, or interim results)		% Premature discontinuation (final results from trials dedicated for cardiovascular safety endpoint)		References (trials not dedicated for cardiovascular safety or interim results)	References (trials dedicated for cardiovascular safety endpoint))
	Active treatment	Control	Active treatment	Control		
**Alogliptin**	/	/	**20.9**	**22.6**	/	White 2013
**Saxagliptin**	/	/	**18.4**	**20.8**	/	Scirica 2013
**Bromocriptin**	**46.8**	**31.7**	/	/	Gaziano 2012	/
**Canagliflozin**	**22.4**	**15.0**	/	/	FDA briefing, Janssen Research & Development 2013	/
**Dapagliflozin**	**12.6**	**15.7**	/	/	FDA briefing, Bristol-Myers Squibb 2011	/
**Exenatide**	**20.8***	**24.9***	/	/	**Kendal 2005; de Fronzo 2005; Buse 2004; Zinman 2007**	/
**Insulin glargine**	**19.3**	**9.1 (cross-over)**	/	/	Investigators of ORIGIN trial, **2012**	/
**Liraglutide**	**15.3**	**19.9**	/	/	**FDA briefing, Novo Nordisk 2009**	/
**Pioglitazone**	**16.4***	**16.6***	/	/	**Dormandy 2009; Wilcox 2008**	/
**Sitagliptin**	**33.5**	**35.2**	/	/	**Williams-Herman 2010**	/

*based on average across multiple study reports; dedicated cardiovascular safety trials data, if available, are given preferential consideration for the choice of noncompliance proportions used in the simulations.
"/" indicates not applicable or data not available

ǂ because different trial recruited different subjects across different countries or regions, and used different study protocols, no direct comparison or conclusions should be made as to the tolerability or quality of trial conduct

Gaziano JM, Cincotta AH, Vinik A, Blonde L, Bohannon N, Scranton R. Effect of bromocriptine-QR (a quick-release formulation of bromocriptine mesylate) on major adverse cardiovascular events in type 2 diabetes subjects. Journal of the American Heart Association. 2012 Oct;1(5):e002279. PubMed PMID: 23316290. Pubmed Central PMCID: 3541616.

FDA briefing, Janssen Research & Development L. Canagliflozin as an Adjunctive Treatment to Diet and Exercise Alone or Co-administered with Other Antihyperglycemic Agents to Improve Glycemic Control in Adults with Type 2 Diabetes Mellitus 2013 [cited 2013 June 9]. Available from: http://www.fda.gov/downloads/AdvisoryCommittees/CommitteesMeetingMaterials/Drugs/EndocrinologicandMetabolicDrugsAdvisoryCommittee/UCM334551.pdf.

FDA briefing, Bristol-Myers Squibb. DAPAGLIFLOZIN, BMS-512148, NDA202293 2011 [cited 2013 June 9]. Available from: http://www.fda.gov/downloads/AdvisoryCommittees/CommitteesMeetingMaterials/Drugs/EndocrinologicandMetabolicDrugsAdvisoryCommittee/UCM262996.pdf.

Scirica BM, Bhatt DL, Braunwald E, et al. Saxagliptin and cardiovascular outcomes in patients with type 2 diabetes mellitus. The New England journal of medicine. 2013; 369: 1317-26.

White WB, Cannon CP, Heller SR, et al. Alogliptin after acute coronary syndrome in patients with type 2 diabetes. The New England journal of medicine. 2013; 369: 1327-35.

Buse JB, Henry RR, Han J, Kim DD, Fineman MS, Baron AD, et al. Effects of exenatide (exendin-4) on glycemic control over 30 weeks in sulfonylurea-treated patients with type 2 diabetes. Diabetes care. 2004 Nov;27(11):2628-35. PubMed PMID: 15504997.

DeFronzo RA, Ratner RE, Han J, Kim DD, Fineman MS, Baron AD. Effects of exenatide (exendin-4) on glycemic control and weight over 30 weeks in metformin-treated patients with type 2 diabetes. Diabetes care. 2005 May;28(5):1092-100. PubMed PMID: 15855572.

Dormandy J, Bhattacharya M, van Troostenburg de Bruyn AR, investigators PR. Safety and tolerability of pioglitazone in high-risk patients with type 2 diabetes: an overview of data from PROactive. Drug safety : an international journal of medical toxicology and drug experience. 2009;32(3):187-202. PubMed PMID: 19338377.

Kendall DM, Riddle MC, Rosenstock J, Zhuang D, Kim DD, Fineman MS, et al. Effects of exenatide (exendin-4) on glycemic control over 30 weeks in patients with type 2 diabetes treated with metformin and a sulfonylurea. Diabetes care. 2005 May;28(5):1083-91. PubMed PMID: 15855571.

Investigators OT, Gerstein HC, Bosch J, Dagenais GR, Diaz R, Jung H, et al. Basal insulin and cardiovascular and other outcomes in dysglycemia. The New England journal of medicine. 2012 Jul 26;367(4):319-28. PubMed PMID: 22686416.

FDA briefing, Novo Nordisk. Liraglutide (injection) for the Treatment of Patients with Type 2 Diabetes 2009 [cited 2013 June 9]. Available from: http://www.fda.gov/downloads/advisorycommittees/committeesmeetingmaterials/drugs/endocrinologicandmetabolicdrugsadvisorycommittee/ucm148659.pdf.

Williams-Herman D, Engel SS, Round E, Johnson J, Golm GT, Guo H, et al. Safety and tolerability of sitagliptin in clinical studies: a pooled analysis of data from 10,246 patients with type 2 diabetes. BMC endocrine disorders. 2010;10:7. PubMed PMID: 20412573. Pubmed Central PMCID: 3161395.

Wilcox R, Kupfer S, Erdmann E, investigators PRS. Effects of pioglitazone on major adverse cardiovascular events in high-risk patients with type 2 diabetes: results from PROspective pioglitAzone Clinical Trial In macro Vascular Events (PROactive 10). American heart journal. 2008 Apr;155(4):712-7. PubMed PMID: 18371481.

Zinman B, Hoogwerf BJ, Duran Garcia S, Milton DR, Giaconia JM, Kim DD, et al. The effect of adding exenatide to a thiazolidinedione in suboptimally controlled type 2 diabetes: a randomized trial. Annals of internal medicine. 2007 Apr 3;146(7):477-85. PubMed PMID: 17404349.

Appendix 3a. Results under all-or-none compliance, summarized across 1,000 simulations under each setting (indexed by Set)

Set	Simulation parameter settings		Estimating	Analysis methods	Summary statistics over 1,000 simulations						Upper bound of 95% confidence interval relative to 1.3, % across 1,000 simulations				
											**ITT**	**≥1.3**	**≥1.3**	**<1.3**	**<1.3**
					**Minimum**	**25th percentile**	**Median**	**75th percentile**	**Maximum**	**Standard deviation**	**Causal**	**≥1.3**	**<1.3**	**≥1.3**	**<1.3**
1	True HR	0.9	HR point estimate	ITT	0.719	0.884	0.931	0.976	1.222	1.084		1.6%	0.0%	16.4%	82.0%
	Compliance type	All-or-none		C-PROPHET	0.616	0.833	0.898	0.963	1.411	1.106					
	% complier	80%	95% upper bound	ITT	0.844	1.036	1.091	1.144	1.433	1.084					
	% never-taker	20%		C-PROPHET	0.758	1.049	1.140	1.234	1.978	1.115					
	RR for MACE never-taker	1	95% lower bound	ITT	0.612	0.754	0.794	0.833	1.042	1.084					
				C-PROPHET	0.506	0.671	0.718	0.768	1.055	1.099					
2	True HR	1	HR point estimate	ITT	0.795	0.944	0.996	1.052	1.278	1.084					
	Compliance type	All-or-none		C-PROPHET	0.750	0.932	0.995	1.066	1.363	1.107	**ITT**	**≥1.3**	**≥1.3**	**<1.3**	**<1.3**
	% complier	80%	95% upper bound	ITT	0.933	1.107	1.167	1.233	1.499	1.084	**Causal**	**≥1.3**	**<1.3**	**≥1.3**	**<1.3**
	% never-taker	20%		C-PROPHET	0.904	1.135	1.215	1.313	1.724	1.116		9.9%	0.0%	**20.8%**	69.3%
	RR for MACE never-taker	1	95% lower bound	ITT	0.678	0.806	0.850	0.897	1.089	1.084					

Set	Simulation parameter settings		Estimating	Analysis methods	Summary statistics over 1,000 simulations						Upper bound of 95% confidence interval relative to 1.3, % across 1,000 simulations				
											**ITT**	**≥1.3**	**≥1.3**	**<1.3**	**<1.3**
					**Minimum**	**25th percentile**	**Median**	**75th percentile**	**Maximum**	**Standard deviation**	**Causal**	**≥1.3**	**<1.3**	**≥1.3**	**<1.3**
				C-PROPHET	0.626	0.771	0.819	0.872	1.099	1.099					
3	True HR	1.1	HR point estimate	ITT	0.824	1.018	1.079	1.147	1.392	1.087					
	Compliance type	All-or-none		C-PROPHET	0.790	1.022	1.098	1.187	1.569	1.110	**ITT**	**≥1.3**	**≥1.3**	**<1.3**	**<1.3**
	% complier	80%	95% upper bound	ITT	0.967	1.193	1.265	1.344	1.635	1.086	**Causal**	**≥1.3**	**<1.3**	**≥1.3**	**<1.3**
	% never-taker	20%		C-PROPHET	0.950	1.248	1.349	1.468	2.035	1.149		39%	0%	26%	35%
	RR for MACE never-taker	1	95% lower bound	ITT	0.703	0.869	0.921	0.978	1.186	1.086					
				C-PROPHET	0.661	0.843	0.900	0.968	1.232	1.117					
4	True HR	1.2	HR point estimate	ITT	0.893	1.096	1.157	1.222	1.501	1.085					
	Compliance type	All-or-none		C-PROPHET	0.870	1.119	1.196	1.282	1.640	1.107	**ITT**	**≥1.3**	**≥1.3**	**<1.3**	**<1.3**
	% complier	80%	95%	ITT	1.047	1.284	1.356	1.433	1.764	1.085	**Causal**	**≥1.3**	**<1.3**	**≥1.3**	**<1.3**
	% never-taker	20%		C-PROPHET	1.053	1.373	1.473	1.590	2.065	1.118		70.9%	0.0%	18.0%	11.1%
	RR for MACE never-taker	1	95% lower bound	ITT	0.762	0.935	0.987	1.042	1.277	1.084					

**Set**	**Simulation parameter settings**		**Estimating**	**Analysis methods**	**Summary statistics over 1,000 simulations**						**Upper bound of 95% confidence interval relative to 1.3, % across 1,000 simulations**				
											**ITT**	**≥1.3**	**≥1.3**	**<1.3**	**<1.3**
					**Minimum**	**25th percentile**	**Median**	**75th percentile**	**Maximum**	**Standard deviation**	**Causal**	**≥1.3**	**<1.3**	**≥1.3**	**<1.3**
				C-PROPHET	0.723	0.919	0.978	1.042	1.316	1.099					
5	True HR	1.3	HR point estimate	ITT	0.962	1.172	1.236	1.307	1.577	1.085					
	Compliance type	All-or-none		C-PROPHET	0.954	1.212	1.296	1.385	1.742	1.108	**ITT**	**≥1.3**	**≥1.3**	**<1.3**	**<1.3**
	% complier	80%	95%	ITT	1.127	1.374	1.450	1.533	1.855	1.086	**Causal**	**≥1.3**	**<1.3**	**≥1.3**	**<1.3**
	% never-taker	20%		C-PROPHET	1.158	1.490	1.600	1.732	2.249	1.119		91.1%	0.0%	6.2%	2.7%
	RR for MACE never-taker	1	95% lower bound	ITT	0.821	1.000	1.054	1.113	1.341	1.085					
				C-PROPHET	0.790	0.993	1.055	1.124	1.391	1.099					
6	True HR	0.9	HR point estimate	ITT	0.710	0.887	0.934	0.985	1.266	1.085					
	Compliance type	All-or-none		C-PROPHET	0.608	0.833	0.902	0.977	1.468	1.130	**ITT**	**≥1.3**	**≥1.3**	**<1.3**	**<1.3**
	% complier	80%	95% upper bound	ITT	0.833	1.039	1.094	1.154	1.485	1.085	**Causal**	**≥1.3**	**<1.3**	**≥1.3**	**<1.3**
	% never-taker	20%		C-PROPHET	0.745	1.053	1.147	1.256	2.016	1.147		0.016	0	0.164	0.820
	RR for MACE never-	2	95% lower	ITT	0.604	0.756	0.797	0.840	1.079	1.085					

Set	Simulation parameter settings		Estimating	Analysis methods	Summary statistics over 1,000 simulations						Upper bound of 95% confidence interval relative to 1.3, % across 1,000 simulations				
											**ITT**	**≥1.3**	**≥1.3**	**<1.3**	**<1.3**
					**Minimum**	**25th percentile**	**Median**	**75th percentile**	**Maximum**	**Standard deviation**	**Causal**	**≥1.3**	**<1.3**	**≥1.3**	**<1.3**
	taker		bound												
				C-PROPHET	0.500	0.671	0.722	0.778	1.110	1.117					
7	True HR	1	HR point estimate	ITT	0.784	0.951	1.002	1.064	1.280	1.086					
	Compliance type	All-or-none		C-PROPHET	0.708	0.929	1.002	1.095	1.471	1.130	**ITT**	**≥1.3**	**≥1.3**	**<1.3**	**<1.3**
	% complier	80%	95% upper bound	ITT	0.920	1.114	1.174	1.247	1.501	1.086	**Causal**	**≥1.3**	**<1.3**	**≥1.3**	**<1.3**
	% never-taker	20%		C-PROPHET	0.872	1.176	1.278	1.419	2.020	1.149		0.094	0	**0.363**	0.543
	RR for MACE never-taker	2	95% lower bound	ITT	0.668	0.811	0.855	0.908	1.091	1.086					
				C-PROPHET	0.574	0.745	0.798	0.867	1.124	1.117					
8	True HR	1.1	HR point estimate	ITT	0.812	1.012	1.066	1.128	1.343	1.082					
	Compliance type	All-or-none		C-PROPHET	0.750	1.018	1.096	1.193	1.573	1.124	**ITT**	**≥1.3**	**≥1.3**	**<1.3**	**<1.3**
	% complier	80%	95% upper bound	ITT	0.952	1.186	1.249	1.323	1.576	1.082	**Causal**	**≥1.3**	**<1.3**	**≥1.3**	**<1.3**
	% never-taker	20%		C-PROPHET	0.921	1.294	1.406	1.549	2.234	1.142		33.0%	0.0%	42.8%	24.2%
	RR for MACE never-	2	95% lower	ITT	0.692	0.864	0.910	0.963	1.144	1.081					

Set	Simulation parameter settings		Estimating	Analysis methods	Summary statistics over 1,000 simulations						Upper bound of 95% confidence interval relative to 1.3, % across 1,000 simulations				
											**ITT**	**≥1.3**	**≥1.3**	**<1.3**	**<1.3**
					**Minimum**	**25th percentile**	**Median**	**75th percentile**	**Maximum**	**Standard deviation**	**Causal**	**≥1.3**	**<1.3**	**≥1.3**	**<1.3**
	taker		bound												
				C-PROPHET	0.616	0.816	0.871	0.943	1.200	1.110					
9	True HR	1.2	HR point estimate	ITT	0.852	1.080	1.135	1.205	1.454	1.085					
	Compliance type	All-or-none		C-PROPHET	0.804	1.116	1.201	1.312	1.793	1.129	**ITT**	**≥1.3**	**≥1.3**	**<1.3**	**<1.3**
	% complier	80%	95% upper bound	ITT	0.999	1.266	1.331	1.413	1.708	1.085	**Causal**	**≥1.3**	**<1.3**	**≥1.3**	**<1.3**
	% never-taker	20%		C-PROPHET	0.990	1.423	1.549	1.708	2.489	1.149		63.5%	0.0%	27.2%	9.3%
	RR for MACE never-taker	2	95% lower bound	ITT	0.727	0.922	0.969	1.028	1.237	1.084					
				C-PROPHET	0.660	0.889	0.952	1.029	1.345	1.114					
10	True HR	1.3	HR point estimate	ITT	0.906	1.142	1.205	1.280	1.538	1.083					
	Compliance type	All-or-none		C-PROPHET	0.876	1.203	1.305	1.423	1.957	1.127	**ITT**	**≥1.3**	**≥1.3**	**<1.3**	**<1.3**
	% complier	80%	95% upper bound	ITT	1.062	1.338	1.413	1.502	1.808	1.084	**Causal**	**≥1.3**	**<1.3**	**≥1.3**	**<1.3**
	% never-taker	20%		C-PROPHET	1.080	1.537	1.683	1.860	2.751	1.147		85.2%	0.0%	12.4%	2.4%
	RR for MACE never-	2	95% lower	ITT	0.773	0.974	1.028	1.091	1.308	1.083					

Set	Simulation parameter settings		Estimating	Analysis methods	Summary statistics over 1,000 simulations						Upper bound of 95% confidence interval relative to 1.3, % across 1,000 simulations				
											**ITT**	**≥1.3**	**≥1.3**	**<1.3**	**<1.3**
					**Minimum**	**25th percentile**	**Median**	**75th percentile**	**Maximum**	**Standard deviation**	**Causal**	**≥1.3**	**<1.3**	**≥1.3**	**<1.3**
	taker		bound												
				C-PROPHET	0.717	0.959	1.028	1.114	1.454	1.112					
11	True HR	0.9	HR point estimate	ITT	0.727	0.890	0.944	0.996	1.197	1.086					
	Compliance type	All-or-none		C-PROPHET	0.585	0.824	0.906	0.993	1.397	1.148	**ITT**	**≥1.3**	**≥1.3**	**<1.3**	**<1.3**
	% complier	60%	95% upper bound	ITT	0.854	1.044	1.106	1.167	1.404	1.086	**Causal**	**≥1.3**	**<1.3**	**≥1.3**	**<1.3**
	% never-taker	40%		C-PROPHET	0.740	1.069	1.193	1.329	2.103	1.175		2.1%	0.0%	30.9%	67.0%
	RR for MACE never-taker	1	95% lower bound	ITT	0.619	0.760	0.805	0.850	1.021	1.086					
				C-PROPHET	0.470	0.649	0.707	0.763	1.025	1.130					
12	True HR	1	HR point estimate	ITT	0.782	0.947	1.003	1.058	1.390	1.086					
	Compliance type	All-or-none		C-PROPHET	0.679	0.915	1.004	1.100	1.909	1.150	**ITT**	**≥1.3**	**≥1.3**	**<1.3**	**<1.3**
	% complier	60%	95% upper bound	ITT	0.918	1.109	1.175	1.240	1.633	1.086	**Causal**	**≥1.3**	**<1.3**	**≥1.3**	**<1.3**
	% never-taker	40%		C-PROPHET	0.854	1.192	1.328	1.477	3.009	1.180		11.0%	0.0%	47.1%	41.9%
	RR for MACE never-	1	95% lower	ITT	0.667	0.808	0.855	0.903	1.184	1.086					

Set	Simulation parameter settings		Estimating	Analysis methods	Summary statistics over 1,000 simulations						Upper bound of 95% confidence interval relative to 1.3, % across 1,000 simulations				
											**ITT**	**≥1.3**	**≥1.3**	**<1.3**	**<1.3**
					**Minimum**	**25th percentile**	**Median**	**75th percentile**	**Maximum**	**Standard deviation**	**Causal**	**≥1.3**	**<1.3**	**≥1.3**	**<1.3**
	taker		bound												
				C-PROPHET	0.547	0.720	0.780	0.844	1.328	1.130					
13	True HR	1.1	HR point estimate	ITT	0.814	1.005	1.061	1.118	1.363	1.084					
	Compliance type	All-or-none		C-PROPHET	0.729	1.007	1.104	1.203	1.759	1.145	**ITT**	**≥1.3**	**≥1.3**	**<1.3**	**<1.3**
	% complier	60%	95% upper bound	ITT	0.954	1.177	1.244	1.310	1.600	1.084	**Causal**	**≥1.3**	**<1.3**	**≥1.3**	**<1.3**
	% never-taker	40%		C-PROPHET	0.917	1.318	1.466	1.627	2.649	1.175		29.0%	0.0%	50.1%	20.9%
	RR for MACE never-taker	1	95% lower bound	ITT	0.694	0.857	0.906	0.954	1.161	1.084					
				C-PROPHET	0.586	0.790	0.854	0.921	1.255	1.126					
14	True HR	1.2	HR point estimate	ITT	0.892	1.063	1.122	1.189	1.472	1.086					
	Compliance type	All-or-none		C-PROPHET	0.839	1.101	1.203	1.329	2.079	1.150	**ITT**	**≥1.3**	**≥1.3**	**<1.3**	**<1.3**
	% complier	60%	95% upper bound	ITT	1.046	1.245	1.315	1.394	1.729	1.086	**Causal**	**≥1.3**	**<1.3**	**≥1.3**	**<1.3**
	% never-taker	40%		C-PROPHET	1.062	1.446	1.603	1.808	3.270	1.182		55.7%	0.0%	36.2%	8.1%
	RR for MACE never-	1	95% lower	ITT	0.761	0.907	0.957	1.014	1.252	1.085					

Set	Simulation parameter settings		Estimating	Analysis methods	Summary statistics over 1,000 simulations						Upper bound of 95% confidence interval relative to 1.3, % across 1,000 simulations				
											**ITT**	**≥1.3**	**≥1.3**	**<1.3**	**<1.3**
					**Minimum**	**25th percentile**	**Median**	**75th percentile**	**Maximum**	**Standard deviation**	**Causal**	**≥1.3**	**<1.3**	**≥1.3**	**<1.3**
	taker		bound												
				C-PROPHET	0.667	0.858	0.929	1.011	1.447	1.129					
15	True HR	1.3	HR point estimate	ITT	0.924	1.119	1.181	1.248	1.622	1.086					
	Compliance type	All-or-none		C-PROPHET	0.891	1.193	1.307	1.428	2.461	1.150	**ITT**	**≥1.3**	**≥1.3**	**<1.3**	**<1.3**
	% complier	60%	95% upper bound	ITT	1.083	1.311	1.385	1.464	1.909	1.086	**Causal**	**≥1.3**	**<1.3**	**≥1.3**	**<1.3**
	% never-taker	40%		C-PROPHET	1.124	1.572	1.751	1.951	3.990	1.184		79%	0%	19%	3%
	RR for MACE never-taker	1	95% lower bound	ITT	0.789	0.954	1.007	1.064	1.378	1.085					
				C-PROPHET	0.716	0.927	1.002	1.086	1.684	1.129					
16	True HR	0.9	HR point estimate	ITT	0.747	0.901	0.957	1.010	1.263	1.085					
	Compliance type	All-or-none		C-PROPHET	0.503	0.788	0.899	1.024	1.953	1.208	**ITT**	**≥1.3**	**≥1.3**	**<1.3**	**<1.3**
	% complier	60%	95% upper bound	ITT	0.877	1.056	1.121	1.183	1.481	1.084	**Causal**	**≥1.3**	**<1.3**	**≥1.3**	**<1.3**
	% never-taker	40%		C-PROPHET	0.691	1.135	1.346	1.604	4.413	1.293		3.1%	0.0%	53.7%	43.2%
	RR for MACE never-	2	95% lower	ITT	0.636	0.769	0.817	0.862	1.076	1.085					

Set	Simulation parameter settings		Estimating	Analysis methods	Summary statistics over 1,000 simulations						Upper bound of 95% confidence interval relative to 1.3, % across 1,000 simulations				
											**ITT**	**≥1.3**	**≥1.3**	**<1.3**	**<1.3**
					**Minimum**	**25th percentile**	**Median**	**75th percentile**	**Maximum**	**Standard deviation**	**Causal**	**≥1.3**	**<1.3**	**≥1.3**	**<1.3**
	taker		bound												
				C-PROPHET	0.382	0.581	0.645	0.720	1.174						
17	True HR	1	HR point estimate	ITT	0.749	0.951	1.003	1.059	1.272	1.082					
	Compliance type	All-or-none		C-PROPHET	0.567	0.893	1.007	1.146	2.133	1.204	**ITT**	**≥1.3**	**≥1.3**	**<1.3**	**<1.3**
	% complier	60%	95% upper bound	ITT	0.879	1.114	1.176	1.241	1.492	1.082	**Causal**	**≥1.3**	**<1.3**	**≥1.3**	**<1.3**
	% never-taker	40%		C-PROPHET	0.745	1.299	1.514	1.811	5.942	1.297		10.0%	0.0%	66.2%	23.8%
	RR for MACE never-taker	2	95% lower bound	ITT	0.638	0.811	0.856	0.903	1.084	1.082					
				C-PROPHET	0.443	0.652	0.721	0.799	1.210	1.161					
18	True HR	1.1	HR point estimate	ITT	0.841	0.990	1.045	1.101	1.332	1.083					
	Compliance type	All-or-none		C-PROPHET	0.705	0.979	1.101	1.244	2.175	1.205	**ITT**	**≥1.3**	**≥1.3**	**<1.3**	**<1.3**
	% complier	60%	95% upper bound	ITT	0.987	1.160	1.224	1.290	1.564	1.083	**Causal**	**≥1.3**	**<1.3**	**≥1.3**	**<1.3**
	% never-taker	40%		C-PROPHET	0.956	1.419	1.665	1.955	5.636	1.293		22.0%	0.0%	64.5%	13.5%
	RR for MACE never-	2	95% lower	ITT	0.718	0.845	0.891	0.939	1.135	1.083					

Set	Simulation parameter settings		Estimating	Analysis methods	Summary statistics over 1,000 simulations						Upper bound of 95% confidence interval relative to 1.3, % across 1,000 simulations				
											**ITT**	**≥1.3**	**≥1.3**	**<1.3**	**<1.3**
					**Minimum**	**25th percentile**	**Median**	**75th percentile**	**Maximum**	**Standard deviation**	**Causal**	**≥1.3**	**<1.3**	**≥1.3**	**<1.3**
	taker		bound												
				C-PROPHET	0.535	0.715	0.788	0.870	1.302	1.163					
19	True HR	1.2	HR point estimate	ITT	0.826	1.030	1.087	1.142	1.364	1.082					
	Compliance type	All-or-none		C-PROPHET	0.703	1.065	1.199	1.345	2.298	1.202	**ITT**	**≥1.3**	**≥1.3**	**<1.3**	**<1.3**
	% complier	60%	95% upper bound	ITT	0.968	1.207	1.274	1.339	1.601	1.083	**Causal**	**≥1.3**	**<1.3**	**≥1.3**	**<1.3**
	% never-taker	40%		C-PROPHET	0.925	1.553	1.829	2.161	5.283	1.291		39.9%	0.0%	54.0%	6.1%
	RR for MACE never-taker	2	95% lower bound	ITT	0.704	0.879	0.927	0.974	1.162	1.082					
				C-PROPHET	0.548	0.775	0.853	0.941	1.372	1.159					
20	True HR	1.3	HR point estimate	ITT	0.864	1.079	1.138	1.197	1.452	1.082					
	Compliance type	All-or-none		C-PROPHET	0.765	1.171	1.315	1.487	2.922	1.206	**ITT**	**≥1.3**	**≥1.3**	**<1.3**	**<1.3**
	% complier	60%	95% upper bound	ITT	1.013	1.265	1.334	1.404	1.706	1.082	**Causal**	**≥1.3**	**<1.3**	**≥1.3**	**<1.3**
	% never-taker	40%		C-PROPHET	1.014	1.726	2.007	2.400	9.513	1.313		61.8%	0.0%	36.4%	1.8%
	RR for MACE never-	2	95% lower	ITT	0.737	0.921	0.971	1.021	1.236	1.081					

Set	Simulation parameter settings		Estimating	Analysis methods	Summary statistics over 1,000 simulations						Upper bound of 95% confidence interval relative to 1.3, % across 1,000 simulations				
											ITT	≥1.3	≥1.3	<1.3	<1.3
					Minimum	25th percentile	Median	75th percentile	Maximum	Standard deviation	Causal	≥1.3	<1.3	≥1.3	<1.3
	taker		bound												
				C-PROPHET	0.594	0.847	0.933	1.028	1.597	1.159					

* only median values are shown, however, mean estimates were almost identical to corresponding medians.

C-PROPHET= complier proportional hazards effect of treatment; ITT=intent-to-treat; HR=hazard ratio; RR=risk ratio; MACE=major adverse cardiovascular events

Table columns in 3 parts: left for parameter settings; middle for distributional statistics of the estimates; right for upper bound comparison percentages.

Appendix 3b. Results under partial noncompliance, with no always-takers, summarized across 1,000 simulations under each setting (indexed by Set)

Set	Simulation parameter settings		Estimating	Analysis methods	Summary statistics over 1,000 simulations						ITT	≥1.3	≥1.3	<1.3	<1.3
					Minimum	25th percentile	Median	75th percentile	Maximum	Standard deviation	SNAFT	≥1.3	<1.3	≥1.3	<1.3
1	True HR	0.9	HR point estimate	ITT	0.709	0.865	0.912	0.965	1.224	1.083		0.6%	0.0%	2.0%	97.4%
	Compliance type	Premature discontinuation		SNAFT	0.672	0.848	0.906	0.961	1.296	1.096					
	% complier	80%	95% upper bound	ITT	0.833	1.014	1.069	1.131	1.435	1.082					
	% never-taker	20%		SNAFT	0.783	1.015	1.079	1.156	1.551	1.097					
	RR for MACE never-taker	1	95% lower bound	ITT	0.604	0.738	0.778	0.824	1.044	1.083					
				SNAFT	0.533	0.697	0.750	0.800	1.062	1.106					
2	True HR	1	HR point estimate	ITT	0.791	0.947	1.001	1.050	1.315	1.081					
	Compliance type	Premature discontinuation		SNAFT	0.741	0.937	1.001	1.060	1.437	1.092	**ITT**	**≥1.3**	**≥1.3**	**<1.3**	**<1.3**
	% complier	80%	95% upper bound	ITT	0.928	1.110	1.173	1.231	1.543	1.081	**SNAFT**	**≥1.3**	**<1.3**	**≥1.3**	**<1.3**
	% never-taker	20%		SNAFT	0.906	1.138	1.218	1.297	1.781	1.099		9.4%	0.3%	11.5 %	78.8%
	RR for MACE never-taker	1	95% lower bound	ITT	0.674	0.808	0.854	0.896	1.121	1.081					
				SNAFT	0.569	0.758	0.817	0.879	1.145	1.099					
3	True HR	1.1	HR point estimate	ITT	0.816	1.036	1.092	1.148	1.375	1.080					
	Compliance type	Premature discontinuation		SNAFT	0.835	1.039	1.102	1.167	1.515	1.091	**ITT**	**≥1.3**	**≥1.3**	**<1.3**	**<1.3**
	% complier	80%	95% upper bound	ITT	0.957	1.214	1.280	1.345	1.614	1.080	**SNAFT**	**≥1.3**	**<1.3**	**≥1.3**	**<1.3**
	% never-taker	20%		SNAFT	0.964	1.245	1.328	1.415	1.898	1.100		43.2 %	0.5%	16.6 %	39.7%
	RR for MACE never-taker	1	95% lower bound	ITT	0.696	0.884	0.932	0.979	1.171	1.080					
				SNAFT	0.700	0.866	0.923	0.978	1.229	1.095					

Set	Simulation parameter settings		Estimating	Analysis methods	Summary statistics over 1,000 simulations						ITT	≥1.3	≥1.3	<1.3	<1.3
					**Minimum**	**25th percentile**	**Median**	**75th percentile**	**Maximum**	**Standard deviation**	**SNAFT**	**≥1.3**	**<1.3**	**≥1.3**	**<1.3**
4	True HR	1.2	HR point estimate	ITT	0.899	1.112	1.181	1.254	1.516	1.087					
	Compliance type	Premature discontinuation		SNAFT	0.885	1.126	1.203	1.289	1.651	1.099	**ITT**	**≥1.3**	**≥1.3**	**<1.3**	**<1.3**
	% complier	80%	95% upper bound	ITT	1.053	1.304	1.385	1.471	1.782	1.087	**SNAFT**	**≥1.3**	**<1.3**	**≥1.3**	**<1.3**
	% never-taker	20%		SNAFT	1.052	1.359	1.456	1.574	2.042	1.109		76.2 %	0.4%	10.6 %	12.8%
	RR for MACE never-taker	1	95% lower bound	ITT	0.767	0.949	1.008	1.069	1.289	1.086					
				SNAFT	0.695	0.945	1.007	1.074	1.352	1.098					
5	True HR	1.3	HR point estimate	ITT	1.002	1.198	1.266	1.335	1.600	1.084					
	Compliance type	Premature discontinuation		SNAFT	1.002	1.219	1.295	1.384	1.758	1.100	**ITT**	**≥1.3**	**≥1.3**	**<1.3**	**<1.3**
	% complier	80%	95% upper bound	ITT	1.174	1.405	1.486	1.567	1.882	1.085	**SNAFT**	**≥1.3**	**<1.3**	**≥1.3**	**<1.3**
	% never-taker	20%		SNAFT	1.160	1.473	1.578	1.696	2.150	1.109		95.1 %	0.3%	2.3%	2.3%
	RR for MACE never-taker	1	95% lower bound	ITT	0.855	1.022	1.080	1.138	1.359	1.084					
				SNAFT	0.808	1.021	1.084	1.151	1.403	1.094					
6	True HR	0.9	HR point estimate	ITT	0.689	0.869	0.918	0.966	1.178	1.083					
	Compliance type	Premature discontinuation		SNAFT	0.587	0.843	0.906	0.961	1.196	1.103	**ITT**	**≥1.3**	**≥1.3**	**<1.3**	**<1.3**
	% complier	80%	95% upper bound	ITT	0.809	1.018	1.076	1.131	1.382	1.083	**SNAFT**	**≥1.3**	**<1.3**	**≥1.3**	**<1.3**
	% never-taker	20%		SNAFT	0.772	1.019	1.094	1.170	1.559	1.105		0.8%	0.0%	3.1%	96.1%
	RR for MACE never-taker	2	95% lower bound	ITT	0.586	0.741	0.784	0.824	1.005	1.084					
				SNAFT	0.479	0.680	0.733	0.789	1.002	1.114					
7	True HR	1	HR point estimate	ITT	0.776	0.949	1.000	1.054	1.290	1.082					

Set	Simulation parameter settings		Estimating	Analysis methods	Summary statistics over 1,000 simulations						ITT	≥1.3	≥1.3	<1.3	<1.3
					Minimum	25th percentile	Median	75th percentile	Maximum	Standard deviation	SNAFT	≥1.3	<1.3	≥1.3	<1.3
	Compliance type	Premature discontinuation		SNAFT	0.736	0.946	1.000	1.059	1.312	1.101	**ITT**	**≥1.3**	**≥1.3**	**<1.3**	**<1.3**
	% complier	80%	95% upper bound	ITT	0.911	1.112	1.172	1.236	1.513	1.082	**SNAFT**	**≥1.3**	**<1.3**	**≥1.3**	**<1.3**
	% never-taker	20%		SNAFT	0.886	1.119	1.188	1.266	1.636	1.107		8.4%	0.0%	16.1 %	75.5%
	RR for MACE never-taker	2	95% lower bound	ITT	0.662	0.810	0.853	0.900	1.099	1.082					
				SNAFT	0.643	0.788	0.840	0.892	1.100	1.099					
8	True HR	1.1	HR point estimate	ITT	0.846	1.025	1.086	1.138	1.448	1.082					
	Compliance type	Premature discontinuation		SNAFT	0.817	1.028	1.098	1.167	1.548	1.101	**ITT**	**≥1.3**	**≥1.3**	**<1.3**	**<1.3**
	% complier	80%	95% upper bound	ITT	0.992	1.201	1.273	1.334	1.701	1.083	**SNAFT**	**≥1.3**	**<1.3**	**≥1.3**	**<1.3**
	% never-taker	20%		SNAFT	0.989	1.261	1.349	1.436	1.945	1.109		37.4 %	0.1%	26.7 %	35.8%
	RR for MACE never-taker	2	95% lower bound	ITT	0.722	0.875	0.927	0.971	1.233	1.082					
				SNAFT	0.622	0.845	0.910	0.965	1.268	1.108					
9	True HR	1.2	HR point estimate	ITT	0.930	1.106	1.169	1.236	1.502	1.083					
	Compliance type	Premature discontinuation		SNAFT	0.909	1.127	1.200	1.283	1.657	1.104	**ITT**	**≥1.3**	**≥1.3**	**<1.3**	**<1.3**
	% complier	80%	95% upper bound	ITT	1.090	1.296	1.370	1.450	1.766	1.084	**SNAFT**	**≥1.3**	**<1.3**	**≥1.3**	**<1.3**
	% never-taker	20%		SNAFT	1.103	1.375	1.470	1.596	2.133	1.114		74.1 %	0.1%	14.9 %	10.9%
	RR for MACE never-taker	2	95% lower bound	ITT	0.794	0.943	0.997	1.054	1.278	1.083					
				SNAFT	0.747	0.930	0.996	1.059	1.331	1.104					
10	True HR	1.3	HR point estimate	ITT	0.984	1.180	1.246	1.317	1.589	1.083					
	Compliance type	Premature discontinuation		SNAFT	0.989	1.213	1.299	1.395	1.861	1.108	**ITT**	**≥1.3**	**≥1.3**	**<1.3**	**<1.3**
	% complier	80%	95% upper bound	ITT	1.153	1.383	1.462	1.545	1.870	1.084	**SNAFT**	**≥1.3**	**<1.3**	**≥1.3**	**<1.3**

**Set**	**Simulation parameter settings**		**Estimating**	**Analysis methods**	**Summary statistics over 1,000 simulations**						**ITT**	**≥1.3**	**≥1.3**	**<1.3**	**<1.3**
					**Minimum**	**25th percentile**	**Median**	**75th percentile**	**Maximum**	**Standard deviation**	**SNAFT**	**≥1.3**	**<1.3**	**≥1.3**	**<1.3**
	% never-taker	20%		SNAFT	1.190	1.488	1.604	1.729	2.258	1.117		92.7%	0.0%	5.0%	2.3%
	RR for MACE never-taker	2	95% lower bound	ITT	0.840	1.006	1.062	1.122	1.351	1.083					
				SNAFT	0.797	1.006	1.070	1.140	1.468	1.100					
11	True HR	0.9	HR point estimate	ITT	0.717	0.871	0.924	0.970	1.230	1.081					
	Compliance type	Premature discontinuation		SNAFT	0.630	0.844	0.908	0.968	1.296	1.106	1.106	**≥1.3**	**≥1.3**	**<1.3**	**<1.3**
	% complier	60%	95% upper bound	ITT	0.842	1.021	1.083	1.137	1.443	1.081	**SNAFT**	**≥1.3**	**<1.3**	**≥1.3**	**<1.3**
	% never-taker	40%		SNAFT	0.762	1.028	1.110	1.187	1.626	1.110		0.6%	0.0%	5.9%	93.5%
	RR for MACE never-taker	1	95% lower bound	ITT	0.611	0.743	0.788	0.828	1.049	1.082					
				SNAFT	0.447	0.673	0.728	0.786	1.062	1.120					
12	True HR	1	HR point estimate	ITT	0.787	0.944	0.994	1.059	1.272	1.087					
	Compliance type	Premature discontinuation		SNAFT	0.728	0.931	0.993	1.070	1.360	1.121	1.113	**≥1.3**	**≥1.3**	**<1.3**	**<1.3**
	% complier	60%	95% upper bound	ITT	0.923	1.107	1.165	1.241	1.492	1.087	**SNAFT**	**≥1.3**	**<1.3**	**≥1.3**	**<1.3**
	% never-taker	40%		SNAFT	0.921	1.136	1.219	1.325	1.790	1.119		10.4%	0.0%	20.9%	68.7%
	RR for MACE never-taker	1	95% lower bound	ITT	0.671	0.806	0.848	0.903	1.084	1.087					
				SNAFT	0.580	0.748	0.806	0.874	1.086	1.121					
13	True HR	1.1	HR point estimate	ITT	0.818	1.017	1.076	1.134	1.409	1.086					
	Compliance type	Premature discontinuation		SNAFT	0.750	1.021	1.097	1.175	1.609	1.113	1.113	**≥1.3**	**≥1.3**	**<1.3**	**<1.3**
	% complier	60%	95% upper bound	ITT	0.960	1.192	1.261	1.329	1.655	1.087	**SNAFT**	**≥1.3**	**<1.3**	**≥1.3**	**<1.3**

Set	Simulation parameter settings		Estimating	Analysis methods	Summary statistics over 1,000 simulations						ITT	≥1.3	≥1.3	<1.3	<1.3
					**Minimum**	**25th percentile**	**Median**	**75th percentile**	**Maximum**	**Standard deviation**	**SNAFT**	**≥1.3**	**<1.3**	**≥1.3**	**<1.3**
	% never-taker	40%		SNAFT	0.963	1.255	1.356	1.455	1.994	1.121		36.1 %	0.0%	28.7 %	35.2%
	RR for MACE never-taker	1	95% lower bound	ITT	0.698	0.868	0.918	0.967	1.200	1.086					
				SNAFT	0.605	0.831	0.896	0.962	1.249	1.116					
14	True HR	1.2	HR point estimate	ITT	0.894	1.095	1.157	1.222	1.502	1.087					
	Compliance type	Premature discontinuation		SNAFT	0.864	1.120	1.197	1.287	1.742	1.112	1.112	**≥1.3**	**≥1.3**	**<1.3**	**<1.3**
	% complier	60%	95% upper bound	ITT	1.048	1.283	1.356	1.434	1.765	1.087	**SNAFT**	**≥1.3**	**<1.3**	**≥1.3**	**<1.3**
	% never-taker	40%		SNAFT	1.062	1.379	1.487	1.608	2.157	1.121		70.6 %	0.0%	18.1 %	11.3%
	RR for MACE never-taker	1	95% lower bound	ITT	0.763	0.934	0.987	1.042	1.278	1.086					
				SNAFT	0.685	0.914	0.985	1.050	1.338	1.112					
15	True HR	1.3	HR point estimate	ITT	0.983	1.173	1.238	1.305	1.648	1.081					
	Compliance type	Premature discontinuation		SNAFT	0.969	1.211	1.303	1.391	1.942	1.108	1.108	**≥1.3**	**≥1.3**	**<1.3**	**<1.3**
	% complier	60%	95% upper bound	ITT	1.152	1.375	1.452	1.531	1.940	1.082	**SNAFT**	**≥1.3**	**<1.3**	**≥1.3**	**<1.3**
	% never-taker	40%		SNAFT	1.181	1.504	1.627	1.747	2.404	1.119		92.4 %	0.0%	5.0%	2.6%
	RR for MACE never-taker	1	95% lower bound	ITT	0.839	1.000	1.056	1.112	1.400	1.081					
				SNAFT	0.753	1.000	1.064	1.133	1.519	1.102					
16	True HR	0.9	HR point estimate	ITT	0.744	0.882	0.932	0.981	1.196	1.083					
	Compliance type	Premature discontinuation		SNAFT	0.603	0.838	0.909	0.974	1.262	1.123	1.123	**≥1.3**	**≥1.3**	**<1.3**	**<1.3**
	% complier	60%	95% upper	ITT	0.873	1.034	1.092	1.150	1.403	1.083	**SNAFT**	**≥1.3**	**<1.3**	**≥1.3**	**<1.3**

Set	Simulation parameter settings		Estimating	Analysis methods	Summary statistics over 1,000 simulations						ITT	≥1.3	≥1.3	<1.3	<1.3
					Minimum	25th percentile	Median	75th percentile	Maximum	Standard deviation	SNAFT	≥1.3	<1.3	≥1.3	<1.3
			bound												
	% never-taker	40%		SNAFT	0.839	1.049	1.135	1.226	1.662	1.124		1.4%	0.0%	10.4 %	88.2%
	RR for MACE never-taker	2	95% lower bound	ITT	0.634	0.753	0.795	0.837	1.020	1.084					
				SNAFT	0.471	0.647	0.706	0.767	1.021	1.142					
17	True HR	1	HR point estimate	ITT	0.778	0.946	0.998	1.059	1.254	1.085					
	Compliance type	Premature discontinuation		SNAFT	0.949	0.622	0.923	0.999	1.080	1.412	1.125	**≥1.3**	**≥1.3**	**<1.3**	**<1.3**
	% complier	60%	95% upper bound	ITT	0.912	1.109	1.170	1.241	1.471	1.085	**SNAFT**	**≥1.3**	**<1.3**	**≥1.3**	**<1.3**
	% never-taker	40%		SNAFT	0.868	1.157	1.261	1.370	1.841	1.131		11.5 %	0.0%	28.1 %	60.4%
	RR for MACE never-taker	2	95% lower bound	ITT	0.663	0.807	0.852	0.904	1.069	1.085					
				SNAFT	0.483	0.722	0.784	0.862	1.090	1.142					
18	True HR	1.1	HR point estimate	ITT	0.813	1.010	1.074	1.133	1.419	1.084					
	Compliance type	Premature discontinuation		SNAFT	0.775	1.018	1.106	1.194	1.582	1.122	1.122	**≥1.3**	**≥1.3**	**<1.3**	**<1.3**
	% complier	60%	95% upper bound	ITT	0.954	1.184	1.259	1.328	1.667	1.085	**SNAFT**	**≥1.3**	**<1.3**	**≥1.3**	**<1.3**
	% never-taker	40%		SNAFT	0.938	1.277	1.398	1.524	2.176	1.135		35.6 %	0.0%	35.0 %	29.4%
	RR for MACE never-taker	2	95% lower bound	ITT	0.693	0.862	0.917	0.967	1.208	1.084					
				SNAFT	0.598	0.805	0.877	0.951	1.271	1.131					
19	True HR	1.2	HR point estimate	ITT	0.881	1.079	1.139	1.206	1.500	1.088					
	Compliance type	Premature discontinuation		SNAFT	0.839	1.104	1.192	1.302	1.781	1.131	1.131	**≥1.3**	**≥1.3**	**<1.3**	**<1.3**

**Set**	**Simulation parameter settings**		**Estimating**	**Analysis methods**	**Summary statistics over 1,000 simulations**						**ITT**	**≥1.3**	**≥1.3**	**<1.3**	**<1.3**
					**Minimum**	**25th percentile**	**Median**	**75th percentile**	**Maximum**	**Standard deviation**	**SNAFT**	**≥1.3**	**<1.3**	**≥1.3**	**<1.3**
	% complier	60%	95% upper bound	ITT	1.033	1.264	1.335	1.414	1.763	1.089	**SNAFT**	**≥1.3**	**<1.3**	**≥1.3**	**<1.3**
	% never-taker	40%		SNAFT	1.018	1.391	1.516	1.664	2.354	1.141		61.5 %	0.0%	27.6 %	10.9%
	RR for MACE never-taker	2	95% lower bound	ITT	0.752	0.920	0.972	1.028	1.276	1.088					
				SNAFT	0.629	0.877	0.953	1.038	1.406	1.135					
20	True HR	1.3	HR point estimate	ITT	0.919	1.147	1.211	1.280	1.623	1.086					
	Compliance type	Premature discontinuation		SNAFT	0.849	1.201	1.302	1.419	2.000	1.128	1.128	**≥1.3**	**≥1.3**	**<1.3**	**<1.3**
	% complier	60%	95% upper bound	ITT	1.077	1.345	1.420	1.502	1.911	1.086	**SNAFT**	**≥1.3**	**<1.3**	**≥1.3**	**<1.3**
	% never-taker	40%		SNAFT	1.085	1.515	1.656	1.811	2.605	1.139		85.1 %	0.0%	12.3 %	2.6%
	RR for MACE never-taker	2	95% lower bound	ITT	0.784	0.978	1.033	1.091	1.379	1.085					
				SNAFT	0.667	0.966	1.043	1.123	1.510	1.124					

* only median values are shown, however, mean estimates were almost identical to corresponding medians.

SNAFT=structural nested accelerated failure time model; ITT=intent-to-treat; HR=hazard ratio; RR=risk ratio; MACE=major adverse cardiovascular events

Table columns in 3 parts: left for parameter settings; middle for distributional statistics of the estimates; right for upper bound comparison percentages.

Appendix 3c. Results when there are always-takers, summarized across 1,000 simulations under each setting (indexed by set)

Set	Simulation parameter settings		Estimating	Analysis methods	Summary statistics over 1,000 simulations						Upper bound of 95% confidence interval relative to 1.3, % across 1,000 simulations				
					Minimum	25th percentile	Median	75th percentile	Maximum	Standard deviations	ITT upper bound	≥1.3	≥1.3	<1.3	<1.3
1	True HR	0.9	HR point estimate	ITT	0.674	0.867	0.918	0.964	1.189	1.081	**Causal upper bound**	**≥1.3**	**<1.3**	**≥1.3**	**<1.3**
	Compliance type	cross-over		SNAFT	0.611	0.853	0.911	0.960	1.209	1.093		1.00%	0.00%	1.80%	97.20%
	% complier	80%	95% upper bound	ITT	0.792	1.016	1.076	1.130	1.394	1.081					
	% never-taker	10%		SNAFT	0.757	1.016	1.080	1.151	1.508	1.096					
	% always-taker	10%	95% lower bound	ITT	0.573	0.739	0.783	0.823	1.014	1.082					
	RR for MACE never-taker	1		SNAFT	0.501	0.703	0.752	0.803	1.030	1.104					
	RR for MACE always-taker	1													
2	True HR	0.9	HR point estimate	ITT	0.665	0.871	0.918	0.968	1.150	1.085		1.00%	0.00%	2.50%	96.50%
	Compliance type	cross-over		SNAFT	0.599	0.853	0.907	0.962	1.220	1.103					
	% complier	80%	95% upper	ITT	0.782	1.021	1.076	1.135	1.348	1.085					

**Set**	**Simulation parameter settings**		**Estimating**	**Analysis methods**	**Summary statistics over 1,000 simulations**						**Upper bound of 95% confidence interval relative to 1.3, % across 1,000 simulations**				
					**Minimum**	**25th percentile**	**Median**	**75th percentile**	**Maximum**	**Standard deviations**	**ITT upper bound**	**≥1.3**	**≥1.3**	**<1.3**	**<1.3**
			bound												
	% never-taker	10%		SNAFT	0.760	1.025	1.093	1.169	1.539	1.105					
	% always-taker	10%	95% lower bound	ITT	0.566	0.743	0.784	0.826	0.981	1.085					
	RR for MACE never-taker	1		SNAFT	0.504	0.694	0.745	0.799	0.971	1.113					
	RR for MACE always-taker	2													
3	True HR	0.9	HR point estimate	ITT	0.694	0.867	0.914	0.964	1.205	1.084		1.10%	0.00%	3.30%	95.60%
	Compliance type	cross-over		SNAFT	0.655	0.845	0.901	0.959	1.246	1.100					
	% complier	80%	95% upper bound	ITT	0.816	1.016	1.071	1.130	1.413	1.084					
	% never-taker	10%		SNAFT	0.792	1.018	1.087	1.161	1.544	1.104					
	% always-taker	10%	95% lower bound	ITT	0.591	0.740	0.779	0.823	1.027	1.085					
	RR for MACE never-taker	2		SNAFT	0.515	0.689	0.740	0.791	1.022	1.110					

Set	Simulation parameter settings		Estimating	Analysis methods	Summary statistics over 1,000 simulations						Upper bound of 95% confidence interval relative to 1.3, % across 1,000 simulations				
					**Minimum**	**25th percentile**	**Median**	**75th percentile**	**Maximum**	**Standard deviations**	**ITT upper bound**	**≥1.3**	**≥1.3**	**<1.3**	**<1.3**
	RR for MACE always-taker	1													
4	True HR	0.9	HR point estimate	ITT	0.719	0.866	0.918	0.965	1.206	1.085		1.00%	0.00%	4.20%	94.80%
	Compliance type	cross-over		SNAFT	0.642	0.843	0.904	0.961	1.222	1.106					
	% complier	80%	95% upper bound	ITT	0.845	1.015	1.076	1.131	1.414	1.085					
	% never-taker	10%		SNAFT	0.768	1.017	1.092	1.169	1.626	1.110					
	% always-taker	10%	95% lower bound	ITT	0.613	0.738	0.783	0.824	1.028	1.085					
	RR for MACE never-taker	2		SNAFT	0.508	0.682	0.732	0.791	1.033	1.117					
	RR for MACE always-taker	2													
5	True HR	1	HR point estimate	ITT	0.796	0.950	1.003	1.059	1.315	1.082		9.70%	0.30%	13.30%	76.70%
	Compliance type	cross-over		SNAFT	0.764	0.946	1.002	1.065	1.338	1.094					
	% complier	80%	95% upper	ITT	0.933	1.114	1.176	1.241	1.543	1.082					

Set	Simulation parameter settings		Estimating	Analysis methods	Summary statistics over 1,000 simulations						Upper bound of 95% confidence interval relative to 1.3, % across 1,000 simulations				
					**Minimum**	**25th percentile**	**Median**	**75th percentile**	**Maximum**	**Standard deviations**	**ITT upper bound**	**≥1.3**	**≥1.3**	**<1.3**	**<1.3**
			bound												
	% never-taker	10%		SNAFT	0.892	1.130	1.206	1.289	1.661	1.102					
	% always-taker	10%	95% lower bound	ITT	0.678	0.811	0.856	0.904	1.120	1.082					
	RR for MACE never-taker	1		SNAFT	0.621	0.784	0.836	0.888	1.109	1.099					
	RR for MACE always-taker	1													
6	True HR	1	HR point estimate	ITT	0.749	0.950	1.002	1.057	1.295	1.082		9.90%	0.00%	15.20%	74.90%
	Compliance type	cross-over		SNAFT	0.735	0.942	1.001	1.063	1.334	1.097					
	% complier	80%	95% upper bound	ITT	0.879	1.113	1.174	1.238	1.520	1.082					
	% never-taker	10%		SNAFT	0.871	1.131	1.212	1.300	1.669	1.105					
	% always-taker	10%	95% lower bound	ITT	0.638	0.810	0.855	0.902	1.104	1.082					
	RR for MACE never-taker	1		SNAFT	0.585	0.773	0.825	0.882	1.109	1.103					

Set	Simulation parameter settings		Estimating	Analysis methods	Summary statistics over 1,000 simulations						Upper bound of 95% confidence interval relative to 1.3, % across 1,000 simulations				
					**Minimum**	**25th percentile**	**Median**	**75th percentile**	**Maximum**	**Standard deviations**	**ITT upper bound**	**≥1.3**	**≥1.3**	**<1.3**	**<1.3**
	RR for MACE always-taker	2													
7	True HR	1	HR point estimate	ITT	0.788	0.945	0.999	1.053	1.345	1.084		9.50%	0.20%	15.10%	75.20%
	Compliance type	cross-over		SNAFT	0.752	0.936	1.000	1.061	1.433	1.100					
	% complier	80%	95% upper bound	ITT	0.925	1.108	1.171	1.234	1.579	1.084					
	% never-taker	10%		SNAFT	0.925	1.127	1.210	1.299	1.763	1.108					
	% always-taker	10%	95% lower bound	ITT	0.672	0.806	0.852	0.899	1.146	1.084					
	RR for MACE never-taker	2		SNAFT	0.598	0.768	0.826	0.885	1.170	1.107					
	RR for MACE always-taker	1													
8	True HR	1	HR point estimate	ITT	0.796	0.947	1.001	1.056	1.344	1.084		9.90%	0.00%	16.00%	74.10%
	Compliance type	cross-over		SNAFT	0.741	0.940	1.000	1.064	1.421	1.104					
	% complier	80%	95% upper	ITT	0.934	1.110	1.173	1.238	1.577	1.084					

Set	Simulation parameter settings		Estimating	Analysis methods	Summary statistics over 1,000 simulations						Upper bound of 95% confidence interval relative to 1.3, % across 1,000 simulations				
					**Minimum**	**25th percentile**	**Median**	**75th percentile**	**Maximum**	**Standard deviations**	**ITT upper bound**	**≥1.3**	**≥1.3**	**<1.3**	**<1.3**
			bound												
	% never-taker	10%		SNAFT	0.909	1.135	1.225	1.303	1.750	1.113					
	% always-taker	10%	95% lower bound	ITT	0.679	0.808	0.854	0.901	1.145	1.084					
	RR for MACE never-taker	2		SNAFT	0.590	0.762	0.822	0.879	1.150	1.112					
	RR for MACE always-taker	2													
9	True HR	1.1	HR point estimate	ITT	0.855	1.033	1.089	1.148	1.434	1.083		41.00%	0.50%	17.40%	41.10%
	Compliance type	cross-over		SNAFT	0.830	1.035	1.098	1.164	1.552	1.095					
	% complier	80%	95% upper bound	ITT	1.002	1.210	1.276	1.346	1.684	1.083					
	% never-taker	10%		SNAFT	1.008	1.238	1.326	1.421	2.025	1.103					
	% always-taker	10%	95% lower bound	ITT	0.729	0.881	0.929	0.979	1.221	1.083					
	RR for MACE never-taker	1		SNAFT	0.684	0.868	0.920	0.980	1.213	1.097					

Set	Simulation parameter settings		Estimating	Analysis methods	Summary statistics over 1,000 simulations						Upper bound of 95% confidence interval relative to 1.3, % across 1,000 simulations				
					**Minimum**	**25th percentile**	**Median**	**75th percentile**	**Maximum**	**Standard deviations**	**ITT upper bound**	**≥1.3**	**≥1.3**	**<1.3**	**<1.3**
	RR for MACE always-taker	1													
10	True HR	1.1	HR point estimate	ITT	0.858	1.031	1.083	1.142	1.413	1.083		38.90%	0.20%	21.90%	39.00%
	Compliance type	cross-over		SNAFT	0.824	1.033	1.094	1.164	1.602	1.099					
	% complier	80%	95% upper bound	ITT	1.006	1.209	1.269	1.338	1.660	1.083					
	% never-taker	10%		SNAFT	1.001	1.251	1.334	1.435	2.031	1.108					
	% always-taker	10%	95% lower bound	ITT	0.732	0.880	0.924	0.974	1.203	1.083					
	RR for MACE never-taker	1		SNAFT	0.671	0.857	0.913	0.970	1.275	1.102					
	RR for MACE always-taker	2													
11	True HR	1.1	HR point estimate	ITT	0.861	1.030	1.084	1.147	1.474	1.084		38.50%	0.70%	22.90%	37.90%
	Compliance type	cross-over		SNAFT	0.833	1.032	1.093	1.171	1.664	1.099					
	% complier	80%	95% upper	ITT	1.009	1.207	1.271	1.345	1.732	1.084					

**Set**	**Simulation parameter settings**		**Estimating**	**Analysis methods**	**Summary statistics over 1,000 simulations**						**Upper bound of 95% confidence interval relative to 1.3, % across 1,000 simulations**				
					**Minimum**	**25th percentile**	**Median**	**75th percentile**	**Maximum**	**Standard deviations**	**ITT upper bound**	**≥1.3**	**≥1.3**	**<1.3**	**<1.3**
			bound												
	% never-taker	10%		SNAFT	1.020	1.252	1.335	1.437	2.040	1.108					
	% always-taker	10%	95% lower bound	ITT	0.734	0.879	0.925	0.979	1.254	1.083					
	RR for MACE never-taker	2		SNAFT	0.680	0.855	0.913	0.973	1.332	1.103					
	RR for MACE always-taker	1													
12	True HR	1.1	HR point estimate	ITT	0.807	1.032	1.090	1.149	1.354	1.081		42.60%	0.30%	23.00%	34.10%
	Compliance type	cross-over		SNAFT	0.739	1.037	1.109	1.179	1.537	1.102					
	% complier	80%	95% upper bound	ITT	0.946	1.209	1.277	1.347	1.589	1.081					
	% never-taker	10%		SNAFT	0.921	1.262	1.358	1.459	1.941	1.110					
	% always-taker	10%	95% lower bound	ITT	0.688	0.880	0.930	0.980	1.153	1.080					
	RR for MACE never-taker	2		SNAFT	0.620	0.852	0.914	0.978	1.210	1.106					

Set	Simulation parameter settings		Estimating	Analysis methods	Summary statistics over 1,000 simulations						Upper bound of 95% confidence interval relative to 1.3, % across 1,000 simulations				
					**Minimum**	**25th percentile**	**Median**	**75th percentile**	**Maximum**	**Standard deviations**	**ITT upper bound**	**≥1.3**	**≥1.3**	**<1.3**	**<1.3**
	RR for MACE always-taker	2													
13	True HR	1.2	HR point estimate	ITT	0.908	1.118	1.181	1.243	1.546	1.085		78.00%	0.40%	8.30%	13.30%
	Compliance type	cross-over		SNAFT	0.908	1.129	1.203	1.279	1.584	1.097					
	% complier	80%	95% upper bound	ITT	1.065	1.311	1.385	1.458	1.818	1.086					
	% never-taker	10%		SNAFT	1.065	1.363	1.459	1.560	2.094	1.107					
	% always-taker	10%	95% lower bound	ITT	0.775	0.954	1.007	1.060	1.314	1.085					
	RR for MACE never-taker	1		SNAFT	0.730	0.943	1.005	1.064	1.339	1.096					
	RR for MACE always-taker	1													
14	True HR	1.2	HR point estimate	ITT	0.887	1.119	1.180	1.242	1.514	1.081		79.60%	0.20%	10.50%	9.70%
	Compliance type	cross-over		SNAFT	0.847	1.134	1.210	1.290	1.699	1.099					
	% complier	80%	95% upper	ITT	1.040	1.312	1.384	1.457	1.780	1.082					

**Set**	**Simulation parameter settings**		**Estimating**	**Analysis methods**	**Summary statistics over 1,000 simulations**						**Upper bound of 95% confidence interval relative to 1.3, % across 1,000 simulations**				
					**Minimum**	**25th percentile**	**Median**	**75th percentile**	**Maximum**	**Standard deviations**	**ITT upper bound**	**≥1.3**	**≥1.3**	**<1.3**	**<1.3**
			bound												
	% never-taker	10%		SNAFT	1.046	1.379	1.479	1.588	2.150	1.109					
	% always-taker	10%	95% lower bound	ITT	0.757	0.955	1.007	1.059	1.288	1.081					
	RR for MACE never-taker	1		SNAFT	0.670	0.948	1.006	1.068	1.328	1.098					
	RR for MACE always-taker	2													
15	True HR	1.2	HR point estimate	ITT	0.880	1.116	1.178	1.238	1.502	1.080		77.10%	0.30%	11.70%	10.90%
	Compliance type	cross-over		SNAFT	0.829	1.129	1.208	1.286	1.691	1.098					
	% complier	80%	95% upper bound	ITT	1.032	1.308	1.381	1.452	1.766	1.080					
	% never-taker	10%		SNAFT	1.032	1.377	1.477	1.578	2.100	1.109					
	% always-taker	10%	95% lower bound	ITT	0.751	0.952	1.005	1.055	1.278	1.080					
	RR for MACE never-taker	2		SNAFT	0.663	0.944	1.003	1.063	1.312	1.098					

Set	Simulation parameter settings		Estimating	Analysis methods	Summary statistics over 1,000 simulations						Upper bound of 95% confidence interval relative to 1.3, % across 1,000 simulations				
					Minimum	25th percentile	Median	75th percentile	Maximum	Standard deviations	ITT upper bound	≥1.3	≥1.3	<1.3	<1.3
	RR for MACE always-taker	1													
16	True HR	1.2	HR point estimate	ITT	0.893	1.102	1.166	1.230	1.495	1.084		72.10%	0.10%	16.30%	11.50%
	Compliance type	cross-over		SNAFT	0.853	1.119	1.203	1.279	1.716	1.106					
	% complier	80%	95% upper bound	ITT	1.047	1.292	1.367	1.443	1.758	1.085					
	% never-taker	10%		SNAFT	1.077	1.372	1.479	1.589	2.159	1.117					
	% always-taker	10%	95% lower bound	ITT	0.762	0.940	0.994	1.049	1.272	1.084					
	RR for MACE never-taker	2		SNAFT	0.720	0.925	0.996	1.057	1.365	1.105					
	RR for MACE always-taker	2													
17	True HR	1.3	HR point estimate	ITT	0.998	1.197	1.264	1.334	1.687	1.083		95.50%	0.00%	1.90%	2.60%
	Compliance type	cross-over		SNAFT	0.999	1.218	1.295	1.380	2.007	1.100					
	% complier	80%	95% upper	ITT	1.169	1.403	1.483	1.566	1.988	1.084					

Set	Simulation parameter settings		Estimating	Analysis methods	Summary statistics over 1,000 simulations						Upper bound of 95% confidence interval relative to 1.3, % across 1,000 simulations				
					**Minimum**	**25th percentile**	**Median**	**75th percentile**	**Maximum**	**Standard deviations**	**ITT upper bound**	**≥1.3**	**≥1.3**	**<1.3**	**<1.3**
			bound												
	% never-taker	10%		SNAFT	1.165	1.470	1.582	1.686	2.430	1.109					
	% always-taker	10%	95% lower bound	ITT	0.851	1.021	1.077	1.137	1.433	1.082					
	RR for MACE never-taker	1		SNAFT	0.804	1.019	1.080	1.146	1.609	1.093					
	RR for MACE always-taker	1													
18	True HR	1.3	HR point estimate	ITT	0.990	1.187	1.253	1.325	1.640	1.083		93.60%	0.10%	3.80%	2.50%
	Compliance type	cross-over		SNAFT	0.983	1.212	1.295	1.383	1.935	1.105					
	% complier	80%	95% upper bound	ITT	1.160	1.392	1.470	1.555	1.930	1.084					
	% never-taker	10%		SNAFT	1.197	1.482	1.598	1.713	2.411	1.115					
	% always-taker	10%	95% lower bound	ITT	0.844	1.012	1.069	1.129	1.393	1.083					
	RR for MACE never-taker	1		SNAFT	0.773	1.012	1.073	1.142	1.562	1.098					

Set	Simulation parameter settings		Estimating	Analysis methods	Summary statistics over 1,000 simulations						Upper bound of 95% confidence interval relative to 1.3, % across 1,000 simulations				
					**Minimum**	**25th percentile**	**Median**	**75th percentile**	**Maximum**	**Standard deviations**	**ITT upper bound**	**≥1.3**	**≥1.3**	**<1.3**	**<1.3**
	RR for MACE always-taker	2													
19	True HR	1.3	HR point estimate	ITT	0.974	1.191	1.262	1.334	1.612	1.086		93.00%	0.10%	5.20%	1.70%
	Compliance type	cross-over		SNAFT	0.978	1.212	1.301	1.399	1.785	1.107					
	% complier	80%	95% upper bound	ITT	1.142	1.396	1.480	1.566	1.897	1.087					
	% never-taker	10%		SNAFT	1.151	1.477	1.597	1.725	2.217	1.116					
	% always-taker	10%	95% lower bound	ITT	0.831	1.015	1.076	1.137	1.370	1.085					
	RR for MACE never-taker	2		SNAFT	0.757	1.016	1.080	1.154	1.446	1.100					
	RR for MACE always-taker	1													
20	True HR	1.3	HR point estimate	ITT	0.941	1.181	1.244	1.322	1.627	1.086		92.20%	0.20%	6.00%	1.60%
	Compliance type	cross-over		SNAFT	0.896	1.212	1.299	1.396	1.848	1.110					
	% complier	80%	95% upper	ITT	1.103	1.384	1.459	1.552	1.915	1.087					

Set	Simulation parameter settings		Estimating	Analysis methods	Summary statistics over 1,000 simulations						Upper bound of 95% confidence interval relative to 1.3, % across 1,000 simulations				
					**Minimum**	**25th percentile**	**Median**	**75th percentile**	**Maximum**	**Standard deviations**	**ITT upper bound**	**≥1.3**	**≥1.3**	**<1.3**	**<1.3**
			bound												
	% never-taker	10%		SNAFT	1.123	1.488	1.609	1.739	2.249	1.121					
	% always-taker	10%	95% lower bound	ITT	0.803	1.007	1.061	1.127	1.383	1.085					
	RR for MACE never-taker	2		SNAFT	0.729	1.007	1.071	1.147	1.499	1.104					
	RR for MACE always-taker	2													
21	True HR	0.9	HR point estimate	ITT	0.727	0.870	0.923	0.970	1.241	1.082		0.70%	0.00%	5.60%	93.70%
	Compliance type	cross-over		SNAFT	0.597	0.840	0.906	0.963	1.379	1.108					
	% complier	60%	95% upper bound	ITT	0.853	1.020	1.081	1.136	1.455	1.082					
	% never-taker	20%		SNAFT	0.833	1.029	1.104	1.188	1.819	1.112					
	% always-taker	20%	95% lower bound	ITT	0.619	0.743	0.787	0.827	1.058	1.082					
	RR for MACE never-taker	1		SNAFT	0.466	0.673	0.728	0.786	1.118	1.119					

Set	Simulation parameter settings		Estimating	Analysis methods	Summary statistics over 1,000 simulations						Upper bound of 95% confidence interval relative to 1.3, % across 1,000 simulations				
					**Minimum**	**25th percentile**	**Median**	**75th percentile**	**Maximum**	**Standard deviations**	**ITT upper bound**	**≥1.3**	**≥1.3**	**<1.3**	**<1.3**
	RR for MACE always-taker	1													
22	True HR	0.9	HR point estimate	ITT	0.740	0.876	0.927	0.977	1.231	1.082		0.90%	0.00%	10.30%	88.80%
	Compliance type	cross-over		SNAFT	0.625	0.838	0.905	0.975	1.356	1.115					
	% complier	60%	95% upper bound	ITT	0.869	1.027	1.086	1.145	1.444	1.082					
	% never-taker	20%		SNAFT	0.805	1.036	1.122	1.217	1.801	1.121					
	% always-taker	20%	95% lower bound	ITT	0.630	0.748	0.791	0.834	1.050	1.082					
	RR for MACE never-taker	1		SNAFT	0.482	0.663	0.722	0.782	1.057	1.126					
	RR for MACE always-taker	2													
23	True HR	0.9	HR point estimate	ITT	0.740	0.872	0.920	0.971	1.195	1.084		1.20%	0.00%	7.60%	91.20%
	Compliance type	cross-over		SNAFT	0.642	0.828	0.894	0.965	1.314	1.118					
	% complier	60%	95% upper	ITT	0.869	1.022	1.079	1.138	1.401	1.084					

Set	Simulation parameter settings		Estimating	Analysis methods	Summary statistics over 1,000 simulations						Upper bound of 95% confidence interval relative to 1.3, % across 1,000 simulations				
					**Minimum**	**25th percentile**	**Median**	**75th percentile**	**Maximum**	**Standard deviations**	**ITT upper bound**	**≥1.3**	**≥1.3**	**<1.3**	**<1.3**
			bound												
	% never-taker	20%		SNAFT	0.808	1.028	1.109	1.196	1.671	1.120					
	% always-taker	20%	95% lower bound	ITT	0.630	0.744	0.785	0.829	1.019	1.085					
	RR for MACE never-taker	2		SNAFT	0.474	0.653	0.709	0.770	1.028	1.130					
	RR for MACE always-taker	1													
24	True HR	0.9	HR point estimate	ITT	0.731	0.876	0.924	0.971	1.180	1.082		1.80%	0.00%	9.20%	89.00%
	Compliance type	cross-over		SNAFT	0.614	0.828	0.893	0.964	1.324	1.120					
	% complier	60%	95% upper bound	ITT	0.858	1.027	1.083	1.138	1.384	1.082					
	% never-taker	20%		SNAFT	0.804	1.038	1.122	1.216	1.709	1.124					
	% always-taker	20%	95% lower bound	ITT	0.622	0.747	0.788	0.828	1.007	1.082					
	RR for MACE never-taker	2		SNAFT	0.442	0.642	0.701	0.760	1.004	1.135					

Set	Simulation parameter settings		Estimating	Analysis methods	Summary statistics over 1,000 simulations						Upper bound of 95% confidence interval relative to 1.3, % across 1,000 simulations				
					**Minimum**	**25th percentile**	**Median**	**75th percentile**	**Maximum**	**Standard deviations**	**ITT upper bound**	**≥1.3**	**≥1.3**	**<1.3**	**<1.3**
	RR for MACE always-taker	2													
25	True HR	1	HR point estimate	ITT	0.744	0.948	0.999	1.054	1.291	1.083		9.20%	0.00%	22.60%	68.20%
	Compliance type	cross-over		SNAFT	0.651	0.934	1.000	1.071	1.412	1.108					
	% complier	60%	95% upper bound	ITT	0.873	1.111	1.171	1.235	1.515	1.083					
	% never-taker	20%		SNAFT	0.843	1.142	1.227	1.326	1.811	1.117					
	% always-taker	20%	95% lower bound	ITT	0.634	0.809	0.853	0.899	1.100	1.083					
	RR for MACE never-taker	1		SNAFT	0.534	0.753	0.809	0.873	1.104	1.116					
	RR for MACE always-taker	1													
26	True HR	1	HR point estimate	ITT	0.769	0.942	0.999	1.053	1.297	1.082		7.80%	0.10%	29.10%	63.00%
	Compliance type	cross-over		SNAFT	0.690	0.926	1.000	1.070	1.436	1.115					
	% complier	60%	95% upper	ITT	0.902	1.104	1.171	1.234	1.521	1.082					

Set	Simulation parameter settings		Estimating	Analysis methods	Summary statistics over 1,000 simulations						Upper bound of 95% confidence interval relative to 1.3, % across 1,000 simulations				
					**Minimum**	**25th percentile**	**Median**	**75th percentile**	**Maximum**	**Standard deviations**	**ITT upper bound**	**≥1.3**	**≥1.3**	**<1.3**	**<1.3**
			bound												
	% never-taker	20%		SNAFT	0.878	1.147	1.244	1.353	1.876	1.125					
	% always-taker	20%	95% lower bound	ITT	0.655	0.804	0.853	0.899	1.105	1.082					
	RR for MACE never-taker	1		SNAFT	0.539	0.737	0.801	0.861	1.141	1.124					
	RR for MACE always-taker	2													
27	True HR	1	HR point estimate	ITT	0.754	0.946	0.998	1.060	1.336	1.086		10.50%	0.00%	25.90%	63.60%
	Compliance type	cross-over		SNAFT	0.680	0.931	0.999	1.078	1.514	1.121					
	% complier	60%	95% upper bound	ITT	0.885	1.109	1.170	1.242	1.568	1.086					
	% never-taker	20%		SNAFT	0.858	1.148	1.248	1.354	1.942	1.129					
	% always-taker	20%	95% lower bound	ITT	0.643	0.807	0.852	0.904	1.138	1.086					
	RR for MACE never-taker	2		SNAFT	0.529	0.737	0.803	0.871	1.217	1.131					

Set	Simulation parameter settings		Estimating	Analysis methods	Summary statistics over 1,000 simulations						Upper bound of 95% confidence interval relative to 1.3, % across 1,000 simulations				
					Minimum	25th percentile	Median	75th percentile	Maximum	Standard deviations	ITT upper bound	≥1.3	≥1.3	<1.3	<1.3
	RR for MACE always-taker	1													
28	True HR	1	HR point estimate	ITT	0.769	0.947	1.002	1.058	1.307	1.085		11.20%	0.00%	29.70%	59.10%
	Compliance type	cross-over		SNAFT	0.684	0.930	1.001	1.077	1.466	1.127					
	% complier	60%	95% upper bound	ITT	0.903	1.109	1.175	1.240	1.534	1.085					
	% never-taker	20%		SNAFT	0.852	1.167	1.262	1.375	1.870	1.135					
	% always-taker	20%	95% lower bound	ITT	0.656	0.808	0.855	0.903	1.114	1.085					
	RR for MACE never-taker	2		SNAFT	0.507	0.734	0.791	0.860	1.181	1.135					
	RR for MACE always-taker	2													
29	True HR	1.1	HR point estimate	ITT	0.842	1.024	1.082	1.145	1.474	1.086		38.30%	0.10%	27.20%	34.40%
	Compliance type	cross-over		SNAFT	0.807	1.027	1.100	1.185	1.815	1.112					
	% complier	60%	95% upper	ITT	0.987	1.200	1.268	1.342	1.732	1.086					

Set	Simulation parameter settings		Estimating	Analysis methods	Summary statistics over 1,000 simulations						Upper bound of 95% confidence interval relative to 1.3, % across 1,000 simulations				
					**Minimum**	**25th percentile**	**Median**	**75th percentile**	**Maximum**	**Standard deviations**	**ITT upper bound**	**≥1.3**	**≥1.3**	**<1.3**	**<1.3**
			bound												
	% never-taker	20%		SNAFT	0.989	1.266	1.365	1.477	2.244	1.123					
	% always-taker	20%	95% lower bound	ITT	0.718	0.873	0.923	0.977	1.254	1.085					
	RR for MACE never-taker	1		SNAFT	0.661	0.840	0.904	0.970	1.356	1.116					
	RR for MACE always-taker	1													
30	True HR	1.1	HR point estimate	ITT	0.842	1.023	1.076	1.135	1.385	1.085		35.60%	0.10%	35.20%	29.10%
	Compliance type	cross-over		SNAFT	0.810	1.026	1.096	1.193	1.707	1.120					
	% complier	60%	95% upper bound	ITT	0.987	1.199	1.261	1.330	1.626	1.085					
	% never-taker	20%		SNAFT	0.983	1.285	1.387	1.509	2.287	1.132					
	% always-taker	20%	95% lower bound	ITT	0.718	0.873	0.918	0.968	1.179	1.085					
	RR for MACE never-taker	1		SNAFT	0.618	0.826	0.891	0.960	1.302	1.122					

Set	Simulation parameter settings		Estimating	Analysis methods	Summary statistics over 1,000 simulations						Upper bound of 95% confidence interval relative to 1.3, % across 1,000 simulations				
					**Minimum**	**25th percentile**	**Median**	**75th percentile**	**Maximum**	**Standard deviations**	**ITT upper bound**	**≥1.3**	**≥1.3**	**<1.3**	**<1.3**
	RR for MACE always-taker	2													
31	True HR	1.1	HR point estimate	ITT	0.836	1.021	1.076	1.134	1.386	1.084		36.20%	0.00%	35.90%	27.90%
	Compliance type	cross-over		SNAFT	0.803	1.025	1.100	1.182	1.665	1.117					
	% complier	60%	95% upper bound	ITT	0.981	1.197	1.261	1.330	1.627	1.085					
	% never-taker	20%		SNAFT	0.979	1.287	1.387	1.505	2.139	1.128					
	% always-taker	20%	95% lower bound	ITT	0.713	0.872	0.918	0.968	1.180	1.084					
	RR for MACE never-taker	2		SNAFT	0.626	0.822	0.892	0.960	1.332	1.121					
	RR for MACE always-taker	1													
32	True HR	1.1	HR point estimate	ITT	0.815	1.017	1.074	1.133	1.404	1.083		34.20%	0.00%	36.00%	29.80%
	Compliance type	cross-over		SNAFT	0.739	1.021	1.100	1.190	1.729	1.122					
	% complier	60%	95% upper	ITT	0.955	1.192	1.259	1.328	1.648	1.083					

Set	Simulation parameter settings		Estimating	Analysis methods	Summary statistics over 1,000 simulations						Upper bound of 95% confidence interval relative to 1.3, % across 1,000 simulations				
					Minimum	25th percentile	Median	75th percentile	Maximum	Standard deviations	ITT upper bound	≥1.3	≥1.3	<1.3	<1.3
			bound												
	% never-taker	20%		SNAFT	0.969	1.281	1.393	1.526	2.183	1.134					
	% always-taker	20%	95% lower bound	ITT	0.695	0.868	0.916	0.966	1.195	1.083					
	RR for MACE never-taker	2		SNAFT	0.574	0.806	0.878	0.951	1.291	1.128					
	RR for MACE always-taker	2													
33	True HR	1.2	HR point estimate	ITT	0.890	1.094	1.149	1.217	1.499	1.084		68.40%	0.20%	18.90%	12.50%
	Compliance type	cross-over		SNAFT	0.883	1.114	1.193	1.281	1.757	1.112					
	% complier	60%	95% upper bound	ITT	1.043	1.282	1.347	1.427	1.762	1.085					
	% never-taker	20%		SNAFT	1.044	1.370	1.485	1.603	2.280	1.123					
	% always-taker	20%	95% lower bound	ITT	0.759	0.933	0.981	1.038	1.275	1.084					
	RR for MACE never-taker	1		SNAFT	0.717	0.907	0.978	1.046	1.351	1.109					

Set	Simulation parameter settings		Estimating	Analysis methods	Summary statistics over 1,000 simulations						Upper bound of 95% confidence interval relative to 1.3, % across 1,000 simulations				
					**Minimum**	**25th percentile**	**Median**	**75th percentile**	**Maximum**	**Standard deviations**	**ITT upper bound**	**≥1.3**	**≥1.3**	**<1.3**	**<1.3**
	RR for MACE always-taker	1													
34	True HR	1.2	HR point estimate	ITT	0.896	1.093	1.149	1.219	1.537	1.083		66.70%	0.10%	23.90%	9.30%
	Compliance type	cross-over		SNAFT	0.872	1.116	1.201	1.305	1.963	1.119					
	% complier	60%	95% upper bound	ITT	1.050	1.282	1.347	1.429	1.807	1.083					
	% never-taker	20%		SNAFT	1.064	1.399	1.524	1.664	2.390	1.132					
	% always-taker	20%	95% lower bound	ITT	0.764	0.933	0.980	1.039	1.307	1.082					
	RR for MACE never-taker	1		SNAFT	0.673	0.903	0.976	1.049	1.483	1.116					
	RR for MACE always-taker	2													
35	True HR	1.2	HR point estimate	ITT	0.901	1.089	1.148	1.220	1.547	1.082		67.80%	0.00%	22.40%	9.80%
	Compliance type	cross-over		SNAFT	0.834	1.114	1.200	1.301	1.835	1.117					
	% complier	60%	95% upper	ITT	1.056	1.276	1.346	1.431	1.820	1.083					

Set	Simulation parameter settings		Estimating	Analysis methods	Summary statistics over 1,000 simulations						Upper bound of 95% confidence interval relative to 1.3, % across 1,000 simulations				
					Minimum	25th percentile	Median	75th percentile	Maximum	Standard deviations	ITT upper bound	≥1.3	≥1.3	<1.3	<1.3
			bound												
	% never-taker	20%		SNAFT	1.075	1.395	1.514	1.653	2.369	1.130					
	% always-taker	20%	95% lower bound	ITT	0.768	0.929	0.980	1.040	1.316	1.082					
	RR for MACE never-taker	2		SNAFT	0.622	0.904	0.973	1.047	1.470	1.116					
	RR for MACE always-taker	1													
36	True HR	1.2	HR point estimate	ITT	0.878	1.085	1.141	1.205	1.471	1.083		65.10%	0.10%	25.60%	9.20%
	Compliance type	cross-over		SNAFT	0.845	1.117	1.200	1.305	1.776	1.125					
	% complier	60%	95% upper bound	ITT	1.030	1.272	1.338	1.413	1.729	1.083					
	% never-taker	20%		SNAFT	1.037	1.413	1.533	1.684	2.229	1.141					
	% always-taker	20%	95% lower bound	ITT	0.749	0.926	0.973	1.027	1.252	1.082					
	RR for MACE never-taker	2		SNAFT	0.643	0.887	0.962	1.035	1.403	1.123					

Set	Simulation parameter settings		Estimating	Analysis methods	Summary statistics over 1,000 simulations						Upper bound of 95% confidence interval relative to 1.3, % across 1,000 simulations				
					**Minimum**	**25th percentile**	**Median**	**75th percentile**	**Maximum**	**Standard deviations**	**ITT upper bound**	**≥1.3**	**≥1.3**	**<1.3**	**<1.3**
	RR for MACE always-taker	2													
37	True HR	1.3	HR point estimate	ITT	0.911	1.165	1.237	1.306	1.647	1.086		89.50%	0.00%	7.90%	2.60%
	Compliance type	cross-over		SNAFT	0.907	1.211	1.297	1.403	1.880	1.116					
	% complier	60%	95% upper bound	ITT	1.068	1.366	1.450	1.533	1.939	1.087					
	% never-taker	20%		SNAFT	1.078	1.503	1.631	1.771	2.436	1.129					
	% always-taker	20%	95% lower bound	ITT	0.778	0.994	1.054	1.113	1.399	1.085					
	RR for MACE never-taker	1		SNAFT	0.722	0.996	1.062	1.141	1.509	1.111					
	RR for MACE always-taker	1													
38	True HR	1.3	HR point estimate	ITT	0.957	1.156	1.222	1.291	1.581	1.084		88.30%	0.00%	9.10%	2.60%
	Compliance type	cross-over		SNAFT	0.937	1.207	1.306	1.405	2.138	1.121					
	% complier	60%	95% upper	ITT	1.122	1.355	1.433	1.514	1.860	1.085					

Set	Simulation parameter settings		Estimating	Analysis methods	Summary statistics over 1,000 simulations						Upper bound of 95% confidence interval relative to 1.3, % across 1,000 simulations				
					**Minimum**	**25th percentile**	**Median**	**75th percentile**	**Maximum**	**Standard deviations**	**ITT upper bound**	**≥1.3**	**≥1.3**	**<1.3**	**<1.3**
			bound												
	% never-taker	20%		SNAFT	1.187	1.520	1.663	1.813	2.795	1.135					
	% always-taker	20%	95% lower bound	ITT	0.817	0.986	1.042	1.100	1.344	1.083					
	RR for MACE never-taker	1		SNAFT	0.736	0.980	1.054	1.127	1.541	1.113					
	RR for MACE always-taker	2													
39	True HR	1.3	HR point estimate	ITT	0.985	1.156	1.222	1.286	1.554	1.083		87.40%	0.00%	11.00%	1.60%
	Compliance type	cross-over		SNAFT	0.970	1.204	1.305	1.407	1.830	1.117					
	% complier	60%	95% upper bound	ITT	1.155	1.355	1.433	1.509	1.827	1.083					
	% never-taker	20%		SNAFT	1.203	1.517	1.653	1.794	2.604	1.129					
	% always-taker	20%	95% lower bound	ITT	0.841	0.986	1.042	1.096	1.321	1.082					
	RR for MACE never-taker	2		SNAFT	0.751	0.979	1.052	1.128	1.489	1.115					

Set	Simulation parameter settings		Estimating	Analysis methods	Summary statistics over 1,000 simulations						Upper bound of 95% confidence interval relative to 1.3, % across 1,000 simulations				
					**Minimum**	**25th percentile**	**Median**	**75th percentile**	**Maximum**	**Standard deviations**	**ITT upper bound**	**≥1.3**	**≥1.3**	**<1.3**	**<1.3**
	RR for MACE always-taker	1													
40	True HR	1.3	HR point estimate	ITT	0.951	1.141	1.206	1.273	1.564	1.083		85.80%	0.00%	12.40%	1.80%
	Compliance type	cross-over		SNAFT	0.939	1.194	1.301	1.416	1.993	1.127					
	% complier	60%	95% upper bound	ITT	1.115	1.338	1.414	1.493	1.840	1.084					
	% never-taker	20%		SNAFT	1.182	1.525	1.680	1.837	2.892	1.144					
	% always-taker	20%	95% lower bound	ITT	0.812	0.974	1.029	1.085	1.330	1.083					
	RR for MACE never-taker	2		SNAFT	0.732	0.963	1.040	1.116	1.548	1.120					
	RR for MACE always-taker	2													

* only median values are shown, however, mean estimates were almost identical to corresponding medians.

ITT=intent-to-treat; HR=hazard ratio; RR=risk ratio; MACE=major adverse cardiovascular events

Table columns in 3 parts: left for parameter settings; middle for distributional statistics of the estimates; right for upper bound comparison percentages.

Appendix 4. STATA® programming codes

Preparations:

If "stcomply" and "strbee" are not already installed in STATA, type the following and follow the links:

net from http://www.mrc-bsu.cam.ac.uk/IW_Stata/

After downloading the "stcomply" package, save it as a separate STATA program file such as "\UserDefinedDirectory\My_stcomply.do", and make the following modification by adding "**matrix psi_e=(`est',`est1',`est2')**" below the "**mkmat psi z, matrix(psi_z)**" statement, which is needed for retrieving the causal hazard ratio estimates during the simulations.
Before simulation programs are run, call it by typing
do "\ UserDefinedDirectory\My_stcomply.do", nostop

Depending on your local settings for STATA, if you receive complains of unbalanced quotation marks when running "My_stcomply.do", check for line breaks, replace ">" with "///" if necessary.

Stata Codes for running simulations, 3 separate modules

Module 1: All-or-none noncompliance

```
 clear all
cd \\ UserDefinedDirectory
set more off
*****************************step 1*********************************
*generate a scaffold dataset for appending N_simul rounds of results
******set up results-saving dataset, do not delete "*scafold*.dta"
*********************************************************************
set obs 1
foreach x in  N N_simul p_trt  p_comply W_shape W_lamda seed HR_CACE HR_never  p_val_ITT {
  gen `x'=99.99
            }
format seed %10.0f
label var N "Sample size, total"
label var N_simul "# of simulations per parameter setting"
label var p_trt "% assigned to trt grp"
label var p_comply "% compliers"
label var W_shape "Weibull shape parameter"
label var W_lamda "Weibull scale param, unexposed complier"
```

```
label var seed "Random seed"
label var HR_CACE "HR:trt effect in compliers"
label var HR_never "HR: never users vs unexposed compliers"
label var p_val_ITT "p-value from ITT Cox model, median of N_simul runs"

foreach y in LULU0000 LULU0001 LULU0010  LULU0011 LULU0100 LULU0101 LULU0110  LULU0111 ///
        LULU1000 LULU1001 LULU1010 LULU1011 LULU1100 LULU1101 LULU1110 LULU1111 {
          gen `y'=0
                                        }
                label var LULU0000 "ITT lower bound<1, upper bound<1.3, RBEE LB<1, UB<1.3"
                label var LULU1111 "ITT 95%LB>=1, UB>=1.3, RBEE (HR__rbee) LB>=1, UB>=1.3"

foreach y in LVLV0000 LVLV0001 LVLV0010  LVLV0011 LVLV0100 LVLV0101 LVLV0110  LVLV0111 ///
        LVLV1000 LVLV1001 LVLV1010 LVLV1011 LVLV1100 LVLV1101 LVLV1110 LVLV1111 {
          gen `y'=0
                                        }
                label var LVLV0000 "ITT lower bound<1, upper bound<1.3, CPROPHET LB<1, VB<1.3"
                label var LVLV1111 "ITT 95%LB>=1, UB>=1.3, CPROPHET (b_psi) LB>=1, VB>=1.3"

foreach b in  b_cox b_cox_l b_cox_u b_psi b_psi_l b_psi_u ///
           HR__rbee HR__rbee_l HR__rbee_u Weib_shp endstudy {
 gen `b'_p25=99.99
 gen `b'_p50=99.99
 gen `b'_p75=99.99
 gen `b'_avg=99.99
 gen `b'_sd=99.99
 gen `b'_min=99.99
 gen `b'_max=99.99

 label var `b'_p25 "25th pctile, `b'"
 label var `b'_p50 "median, `b'"
 label var `b'_p75 "75th pctile, `b'"
 label var `b'_avg "mean, `b'"
 label var `b'_sd "SD, `b'"
 label var `b'_min "min, `b'"
 label var `b'_max "max, `b'"
 }
save scafold_CPH, replace

*******************step 2***********************
```

```
*set up data frame for running each 1 simulation

quietly {
clear all
		do "\UserDefinedDirectory ", nostop

		***specify sample size (10000)
		set obs 10000
		scalar N=10000

		gen id=_n
		scalar p_trt=0.5
		scalar n_trt=N*p_trt
**in this example, % of compliers is 60%, change this to 0.8 if 80% compliers, and so on
		scalar p_comply=0.6
		scalar W_shape=1.223548
		scalar W_lamda=0.02470739

		gen R=_n<=n_trt
		gen D=_n<=p_comply*n_trt

		gen complier=_n<=p_comply*n_trt
		replace complier=1 if _n >n_trt & _n<=n_trt+(N-n_trt)*p_comply

		gen xo=1 if complier==0 & R==1

		foreach x in t_pot  xoyrs t MACE  endFU {
				gen `x'=.
																}
		replace xoyrs=0 if complier==0 & R==1

save frame_CPH, replace
*closes quietly loop above
 }
 scalar seed=28431754
 set seed 28431754
***Alternative seeds included: 822246, 2202880

********step 3: run simulation for N_simul times, each time using tempframe_perfect.dta*******
 ***Step 3a: simulation runs N_simul times on data frame
```

```
  nois _dots 0, title(Loop running) reps(5)
tempname sim
global CACE = 90
display "program begins on " c(current_date) " at "   c(current_time)
while $CACE<=130  {

 global Never=50
   while $Never<=200 {
		nois _dots `i' 0
			use frame_CPH, clear
			scalar HR_CACE=$CACE/100
			scalar HR_never=$Never/100

		postfile `sim' b_cox b_cox_l b_cox_u p_val_Cox psi psi_l psi_u ///
			HR__rbee HR__rbee_l HR__rbee_u Weib_shp endstudy using results, replace
 quietly {
   scalar N_simul=1000
		forvalues i = 1/1000 {
		replace t_pot=(-ln(uniform())/W_lamda)^(1/W_shape) if complier==1 & R==0
		replace t_pot=(-ln(uniform())/(HR_CACE*W_lamda))^(1/W_shape) if complier==1 & R==1
		replace t_pot=(-ln(uniform())/(HR_never*W_lamda))^(1/W_shape) if complier==0
		**this example simulated event-driven type of study, which ended when there were 611 MACE
***for fixed-length study, specify endstudy=3, for example if administrative censoring occurs at year 3
		centile t_pot, centile(6.11)

		scalar endstudy=r(c_1)
		replace endFU=endstudy
		replace MACE=t_pot<=endFU
		replace t=min(t_pot,endFU)

		stset t, fail(MACE)

**ITT: Cox model HR estimate (natural-log-scale)
		stcox R, nolog
			 matrix b=e(b)
			 matrix V=e(V)
			scalar b=b[1,1]
			scalar sd=sqrt(V[1,1])
			scalar b_cox=b
```

```
                scalar b_cox_l=b-invnorm(0.975)*sd
                scalar b_cox_u=b+invnorm(0.975)*sd
                scalar p_val_Cox= chi2tail(1,e(chi2))

**CPROPHET: causal proportional hazards model
                 replace D=. if R==0
                mystcomp R D, data
                scalar psi=psi_e[1,1]
                scalar psi_l=psi_e[1,2]
                scalar psi_u=psi_e[1,3]


**ITT: Weibull model HR estimate (natural-log-scale)
                streg R, dist(weib)
                **also estimates Shape parameter
                scalar Weib_shp=e(aux_p)
                *matrix b=e(b)
                *matrix V=e(V)
                *scalar b=b[1,1]
                *scalar sd=sqrt(V[1,1])
                *scalar b_Weib=b
                *scalar b_Weib_l=b-invnorm(0.975)*sd
                *scalar b_Weib_u=b+invnorm(0.975)*sd

**Causal: time ratio (TR=exp(-psi) comparing exposed vs unexposed)
  **here psi=[-Ln(TR)], i.e, on natural log scale, recensoring applied
                strbee R, xo1( xoyrs xo)   endstudy(endFU)
                scalar rbee=r(psi)
                scalar rbee_l=r(psilow)
                scalar rbee_u=r(psiupp)

                **convert TR to HR (natural-log-scale)
                *Weibull model stimated shape parameter above Weib_shp, not pre-specified parameter W_shape
                **note: HR=TR^(-W_shape)=exp(-rbee)^(-W_shape),
          **note:  ln(HR)=-W_shape*ln(TR)=(-W_shape)*(-rbee)=W_shape*rbee

                scalar HR__rbee=rbee*Weib_shp
                scalar HR__rbee_l=rbee_l*Weib_shp
                scalar HR__rbee_u=rbee_u*Weib_shp
```

```
post `sim' (b_cox) (b_cox_l) (b_cox_u) (p_val_Cox) (psi) (psi_l) (psi_u) ///
             (HR__rbee) (HR__rbee_l) (HR__rbee_u)  (Weib_shp)  (endstudy)
*closes simulation loop of 1000 runs
}
*closes quietly
}
postclose `sim'

***Step 3b: summarize results of the N_simul simulations under each 1 parameter setting
use results, clear

 foreach b in  b_cox b_cox_l b_cox_u HR__rbee HR__rbee_l HR__rbee_u Weib_shp endstudy {
                                                                                                                                   sum `b', d
                                                scalar `b'_p25=r(p25)                                                                              scalar
`b'_p50=r(p50)                                                                         scalar `b'_p75=r(p75)
                          scalar `b'_avg=r(mean)                                                                                   scalar `b'_sd= r(sd)
                                                      scalar `b'_min= r(min)                                                                              scalar
`b'_max= r(max)
                                                                                                                  }
        sum p_val_Cox, d
                  scalar p_val_ITT=r(p50)

                  count if b_cox_l<0 & b_cox_u<ln(1.3) & HR__rbee_l<0 & HR__rbee_u<ln(1.3)
           scalar LULU0000=r(N)/N_simul
           count if b_cox_l<0 & b_cox_u<ln(1.3) & HR__rbee_l<0 & HR__rbee_u>=ln(1.3)
           scalar LULU0001=r(N)/N_simul
           count if b_cox_l<0 & b_cox_u<ln(1.3) & HR__rbee_l>=0 & HR__rbee_u<ln(1.3)
           scalar LULU0010=r(N)/N_simul
           count if b_cox_l<0 & b_cox_u<ln(1.3) & HR__rbee_l>=0 & HR__rbee_u>=ln(1.3)
           scalar LULU0011=r(N)/N_simul

           count if b_cox_l<0 & b_cox_u>=ln(1.3) & HR__rbee_l<0 & HR__rbee_u<ln(1.3)
           scalar LULU0100=r(N)/N_simul
           count if b_cox_l<0 & b_cox_u>=ln(1.3) & HR__rbee_l<0 & HR__rbee_u>=ln(1.3)
           scalar LULU0101=r(N)/N_simul
           count if b_cox_l<0 & b_cox_u>=ln(1.3) & HR__rbee_l>=0 & HR__rbee_u<ln(1.3)
           scalar LULU0110=r(N)/N_simul
           count if b_cox_l<0 & b_cox_u>=ln(1.3) & HR__rbee_l>=0 & HR__rbee_u>=ln(1.3)
           scalar LULU0111=r(N)/N_simul
```

```
count if b_cox_l>=0 & b_cox_u<ln(1.3) & HR__rbee_l<0 & HR__rbee_u<ln(1.3)
scalar LULU1000=r(N)/N_simul
count if b_cox_l>=0 & b_cox_u<ln(1.3) & HR__rbee_l<0 & HR__rbee_u>=ln(1.3)
scalar LULU1001=r(N)/N_simul
count if b_cox_l>=0 & b_cox_u<ln(1.3) & HR__rbee_l>=0 & HR__rbee_u<ln(1.3)
scalar LULU1010=r(N)/N_simul
count if b_cox_l>=0 & b_cox_u<ln(1.3) & HR__rbee_l>=0 & HR__rbee_u>=ln(1.3)
scalar LULU1011=r(N)/N_simul

count if b_cox_l>=0 & b_cox_u>=ln(1.3) & HR__rbee_l<0 & HR__rbee_u<ln(1.3)
scalar LULU1100=r(N)/N_simul
count if b_cox_l>=0 & b_cox_u>=ln(1.3) & HR__rbee_l<0 & HR__rbee_u>=ln(1.3)
scalar LULU1101=r(N)/N_simul
count if b_cox_l>=0 & b_cox_u>=ln(1.3) & HR__rbee_l>=0 & HR__rbee_u<ln(1.3)
scalar LULU1110=r(N)/N_simul
count if b_cox_l>=0 & b_cox_u>=ln(1.3) & HR__rbee_l>=0 & HR__rbee_u>=ln(1.3)
scalar LULU1111=r(N)/N_simul

**summary should be done on the natural log scale
foreach HR in  psi psi_l psi_u {
        gen b_`HR'=ln(`HR')
        sum b_`HR', d
        scalar b_`HR'_p25=r(p25)
        scalar b_`HR'_p50=r(p50)
        scalar b_`HR'_p75=r(p75)
        scalar b_`HR'_avg=r(mean)
        scalar b_`HR'_sd= r(sd)
        scalar b_`HR'_min= r(min)
        scalar b_`HR'_max= r(max)
                                        }
        count if b_cox_l<0 & b_cox_u<ln(1.3) & b_psi_l<0 & b_psi_u<ln(1.3)
scalar LVLV0000=r(N)/N_simul
count if b_cox_l<0 & b_cox_u<ln(1.3) & b_psi_l<0 & b_psi_u>=ln(1.3)
scalar LVLV0001=r(N)/N_simul
count if b_cox_l<0 & b_cox_u<ln(1.3) & b_psi_l>=0 & b_psi_u<ln(1.3)
scalar LVLV0010=r(N)/N_simul
count if b_cox_l<0 & b_cox_u<ln(1.3) & b_psi_l>=0 & b_psi_u>=ln(1.3)
scalar LVLV0011=r(N)/N_simul
```

```
        count if b_cox_l<0 & b_cox_u>=ln(1.3) & b_psi_l<0 & b_psi_u<ln(1.3)
        scalar LVLV0100=r(N)/N_simul
        count if b_cox_l<0 & b_cox_u>=ln(1.3) & b_psi_l<0 & b_psi_u>=ln(1.3)
        scalar LVLV0101=r(N)/N_simul
        count if b_cox_l<0 & b_cox_u>=ln(1.3) & b_psi_l>=0 & b_psi_u<ln(1.3)
        scalar LVLV0110=r(N)/N_simul
        count if b_cox_l<0 & b_cox_u>=ln(1.3) & b_psi_l>=0 & b_psi_u>=ln(1.3)
        scalar LVLV0111=r(N)/N_simul

        count if b_cox_l>=0 & b_cox_u<ln(1.3) & b_psi_l<0 & b_psi_u<ln(1.3)
        scalar LVLV1000=r(N)/N_simul
        count if b_cox_l>=0 & b_cox_u<ln(1.3) & b_psi_l<0 & b_psi_u>=ln(1.3)
        scalar LVLV1001=r(N)/N_simul
        count if b_cox_l>=0 & b_cox_u<ln(1.3) & b_psi_l>=0 & b_psi_u<ln(1.3)
        scalar LVLV1010=r(N)/N_simul
        count if b_cox_l>=0 & b_cox_u<ln(1.3) & b_psi_l>=0 & b_psi_u>=ln(1.3)
        scalar LVLV1011=r(N)/N_simul

        count if b_cox_l>=0 & b_cox_u>=ln(1.3) & b_psi_l<0 & b_psi_u<ln(1.3)
        scalar LVLV1100=r(N)/N_simul
        count if b_cox_l>=0 & b_cox_u>=ln(1.3) & b_psi_l<0 & b_psi_u>=ln(1.3)
        scalar LVLV1101=r(N)/N_simul
        count if b_cox_l>=0 & b_cox_u>=ln(1.3) & b_psi_l>=0 & b_psi_u<ln(1.3)
        scalar LVLV1110=r(N)/N_simul
        count if b_cox_l>=0 & b_cox_u>=ln(1.3) & b_psi_l>=0 & b_psi_u>=ln(1.3)
        scalar LVLV1111=r(N)/N_simul

clear
****step 3c:save summary of N_simul simulations into 1 temporary dataset for appending
set obs 1

        foreach vn in  N N_simul p_trt n_trt p_comply W_shape W_lamda seed HR_CACE HR_never  ///
                        LULU0000 LULU0001 LULU0010  LULU0011 LULU0100 LULU0101 LULU0110  LULU0111 ///
       LULU1000 LULU1001 LULU1010 LULU1011 LULU1100 LULU1101 LULU1110 LULU1111 ///
                        LVLV0000 LVLV0001 LVLV0010  LVLV0011 LVLV0100 LVLV0101 LVLV0110  LVLV0111 ///
       LVLV1000 LVLV1001 LVLV1010 LVLV1011 LVLV1100 LVLV1101 LVLV1110 LVLV1111 p_val_ITT {
                                        gen `vn'=`vn'
                                            }

        foreach sn in  b_cox b_cox_l b_cox_u b_psi b_psi_l b_psi_u  ///
```

```
	HR__rbee HR__rbee_l HR__rbee_u Weib_shp endstudy  {

					gen `sn'_p25=`sn'_p25
					gen `sn'_p50=`sn'_p50
					gen `sn'_p75=`sn'_p75
					gen `sn'_avg=`sn'_avg
					gen `sn'_sd=`sn'_sd
					gen `sn'_min= `sn'_min
					gen `sn'_max= `sn'_max
												}

save temp_drop, replace
use scafold_CPH, clear
append using temp_drop
save scafold_CPH, replace

*close Never loop
global Never=$Never+50
}
*close CACE loop

global CACE=$CACE+10
}

display "ended  on " c(current_date) " at "   c(current_time)


*********step 4: post-simulation summary, all parameter settings*************
***********************exponentiate log-scale coefficients******************
save exponentiated, replace
drop if HR_CACE==float(99.99)
foreach b in  b_cox b_cox_l b_cox_u b_psi b_psi_l b_psi_u HR__rbee HR__rbee_l HR__rbee_u {
				replace `b'_p25=exp(`b'_p25)
				replace `b'_p50=exp(`b'_p50)
				replace `b'_p75=exp(`b'_p75)
				replace `b'_avg=exp(`b'_avg)
				replace `b'_sd=exp( `b'_sd)
				replace `b'_min=exp(`b'_min)
				replace `b'_max=exp(`b'_max)
								}
```

```
save exponentiated, replace

use exponentiated, clear
* format b* %4.2f


gen UU00=LULU0000+LULU1010+LULU0010+LULU1000
gen UU01=LULU0001+LULU1001+LULU0011+LULU1011
gen UU10=LULU0100+LULU0110+LULU1100+LULU1110
gen UU11=LULU1111+LULU0101+LULU0111+LULU1101
label var UU00 "95%UB: ITT, rbee HR both<1.3"
label var UU01 "95% UB:ITT<1.3, rbee HR >=1.3"
label var UU11 "95%UB: ITT and rbee HR both>=1.3"
label var UU10 "95%UB: ITT>=1.3 rbee HR<1.3"

gen L0UU00=LULU0000+LULU0010
gen L0UU01=LULU0001+LULU0011
gen L0UU10=LULU0100+LULU0110
gen L0UU11=LULU0101+LULU0111

label var L0UU00 "95%UB:ITT,rbee HR both<1.3; ITT LB<1"
label var L0UU01 "95% UB:ITT<1.3, rbee HR >=1.3; ITT LB<1"
label var L0UU11 "95%UB: ITT and rbee HR both>=1.3; ITT LB<1"
label var L0UU10 "95%UB: ITT>=1.3 rbee HR<1.3; ITT LB<1"

gen L1UU00=UU00-L0UU00
gen L1UU01=UU01-L0UU01
gen L1UU10=UU10-L0UU10
gen L1UU11=UU11-L0UU11

label var L1UU00 "95%UB:ITT,rbee HR both<1.3; ITT LB>=1"
label var L1UU01 "95% UB:ITT<1.3, rbee HR >=1.3; ITT LB>=1"
label var L1UU11 "95%UB: ITT and rbee HR both>=1.3; ITT LB>=1"
label var L1UU10 "95%UB: ITT>=1.3 rbee HR<1.3; ITT LB>=1"


gen UV00=LVLV0000+LVLV1010+LVLV0010+LVLV1000
gen UV01=LVLV0001+LVLV1001+LVLV0011+LVLV1011
gen UV10=LVLV0100+LVLV0110+LVLV1100+LVLV1110
gen UV11=LVLV1111+LVLV0101+LVLV0111+LVLV1101
```

```
*CPH=causal proportional-hazards
label var UV00 "95%UB: ITT, CPH HR both<1.3"
label var UV01 "95% UB:ITT<1.3, CPH HR >=1.3"
label var UV11 "95%UB: ITT and CPH HR both>=1.3"
label var UV10 "95%UB: ITT>=1.3 CPH HR<1.3"

gen L0UV00=LVLV0000+LVLV0010
gen L0UV01=LVLV0001+LVLV0011
gen L0UV10=LVLV0100+LVLV0110
gen L0UV11=LVLV0101+LVLV0111

label var L0UV00 "95%UB:ITT,CPH HR both<1.3; ITT LB<1"
label var L0UV01 "95% UB:ITT<1.3, CPH HR >=1.3; ITT LB<1"
label var L0UV11 "95%UB: ITT and CPH HR both>=1.3; ITT LB<1"
label var L0UV10 "95%UB: ITT>=1.3 CPH HR<1.3; ITT LB<1"

gen L1UV00=UV00-L0UV00
gen L1UV01=UV01-L0UV01
gen L1UV10=UV10-L0UV10
gen L1UV11=UV11-L0UV11

label var L1UV00 "95%UB:ITT,CPH HR both<1.3; ITT LB>=1"
label var L1UV01 "95% UB:ITT<1.3, CPH HR >=1.3; ITT LB>=1"
label var L1UV11 "95%UB: ITT and CPH HR both>=1.3; ITT LB>=1"
label var L1UV10 "95%UB: ITT>=1.3 CPH HR<1.3; ITT LB>=1"


save exponentiated, replace
use exponentiated, clear
sort HR_CACE HR_never b_cox_avg
format seed %10.0f
**wide output for bar charts comparing Upper bound % relative to 1.3
order HR_CACE HR_never  UU11 UU10 UU01 UU00 L0UU11 L0UU01 L0UU00 L1UU11 L1UU01 L1UU00 ///
                                   UV11 UV01 UV00 L0UV11 L0UV01 L0UV00 L1UV11 L1UV01 L1UV00, first
bro
*the following re-shaping of results makes it convenient to view parameters for box-plots
reshape long b_cox b_psi HR__rbee b_cox_u b_psi_u   HR__rbee_u b_cox_l b_psi_l HR__rbee_l , i(HR_CACE HR_never) j(param) string
**long output for box plots
order HR_CACE HR_never param b_cox b_psi HR__rbee b_cox_u b_psi_u   HR__rbee_u b_cox_l b_psi_l HR__rbee_l, first
```

```
*sort hr_cace hr_never b_cox
sort HR_CACE HR_never b_cox
bro
des, replace
bro name varlab
*******end of program*******
```

Module 2: Partial compliance, no always-takers

```
clear all
set more off
cd \\UserDefinedDirectory
**This program simulates the simulation of no always-takers, premature discontinuation

**note: HR=TR^(-W_shape), log(HR)=-W_shape*log(TR)
********************step 1************************
*generate a scaffold dataset for appending N_simul rounds of results
set obs 1
foreach x in  N N_simul p_trt  p_comply  W_shape W_lamda  ///
        HR_CACE HR_never  TR  seed    {
                                    gen `x'=99.99
                                                                                }
                label var N "==Total sample size, randomized, treatment+ctrl"
                label var N_simul "#simulations for each unique parameter combo"
                label var p_trt "==% assigned to treatment arm"
                label var p_comply "==% compliers, same across 2 arms by randomization"
                *label var p_never "==% never-taker, same across 2 arms by randomization"
                *label var p_always "==%always-taker=1-%compliers-%never-takers"
                label var W_shape "==Weibull shape param,based on ADVANCE+Framingham, fixed"
                label var W_lamda "==Weibull scale param, unexposed complier"

                label var HR_CACE "==HR:trt effect in compliers"
                label var HR_never "==HR: never-users vs unexposed compliers"
                *label var HR_always "==HR: always-takers vs unexposed compliers"
                label var TR "==Causal survival time ratio: exposed vs unexposed compliers"
                label var seed "Random seed"


foreach y in LULU0000 LULU0001 LULU0010  LULU0011 LULU0100 LULU0101 LULU0110  LULU0111 ///
        LULU1000 LULU1001 LULU1010 LULU1011 LULU1100 LULU1101 LULU1110 LULU1111 {
```

```
                gen `y'=0
                                                    }
                        label var LULU0000 "ITT lower bound<1, upper bound<1.3, causal LB<1, UB<1.3"
                        label var LULU1111 "ITT 95%LB>=1, UB>=1.3, causal LB>=1, UB>=1.3"

foreach b in  ITT ITT_l ITT_u psi psi_l psi_u b_cox b_cox_l b_cox_u ///
           b_Weib b_Weib_l b_Weib_u Weib_shp HR__psi HR__psi_l HR__psi_u ///
                                       HR__ITT HR__ITT_l HR__ITT_u  endstudy {
  gen `b'_p25=99.99
  gen `b'_p50=99.99
  gen `b'_p75=99.99
  gen `b'_avg=99.99
  gen `b'_sd=99.99
  gen `b'_min=99.99
  gen `b'_max=99.99

  label var `b'_p25 "25th pctile, `b'"
  label var `b'_p50 "median, `b'"
  label var `b'_p75 "75th pctile, `b'"

  label var `b'_sd "SD, `b'"
  label var `b'_min "min, `b'"
  label var `b'_max "max, `b'"
  }
label var ITT_avg "mean, point est ITT-TR(rbee ignoring xo)"
label var ITT_l_avg "mean, LCL of ITT-TR"
label var ITT_u_avg "mean, UCL of ITT-TR"
label var psi_avg "mean, point est psi-TR(rbee handling xo)"
label var psi_l_avg "mean, LCL of psi-TR"
label var b_cox_avg "mean, point est ITT-Cox-HR"
label var b_Weib_avg "mean, point est ITT-Weibull HR"
label var HR__psi_avg "mean, point est psi-HR(psi-TR^(-Weib_shp))"
label var HR__ITT_avg "mean, point est ITT-HR(ITT-TR^(-Weib_shp"
save scafold, replace
clear all

*******************step 2**********************
*set up data frame for running each 1 simulation
quietly {
```

```
***specify sample size (10000)
        set obs 10000
        scalar N=10000

        gen id=_n
        scalar p_trt=0.5
        scalar n_trt=N*p_trt
        scalar p_comply=0.6
        gen R=_n<=n_trt
        gen D=_n<=p_comply*n_trt

        gen complier=_n<=p_comply*n_trt
        replace complier=1 if _n >n_trt & _n<=n_trt+(N-n_trt)*p_comply

        gen xo=1 if complier==0 & R==1

        foreach x in t_pot  U0 U1 xoyrs t MACE  endFU {
                gen `x'=.
                                                        }

        ***in theory, all N subjects will have MACE event when FU through largest potential life-time t_pot
        ***percentile of t_pot to end FU/admin censor when 611 MACE events occur (90%power to exclude UB 1.3)
        scalar EvntDriven=611*100/N
        **allocation ratio


        scalar seed=822246
        set seed 822246

save frame_noAT, replace
*closes quietly loop
}

********step 3: run simulation for N_simul times, each time using frame_noAT.dta*******
 ***Step 3a: simulation runs N_simul times on data frame
 nois _dots 0, title(Loop running) reps(5)
tempname sim
scalar W_shape=1.223548
scalar W_lamda=0.02470739
```

```
global CACE =80
display "program begins on " c(current_date) " at "   c(current_time)
while $CACE<=130  {

global Never=50
   while $Never<=200 {
              nois _dots `i' 0
   use frame_noAT, clear
                         scalar HR_CACE=$CACE/100

                         scalar HR_never=$Never/100
                         *scalar HR_always=HR_never

                         scalar HR_always=100/$Never
                         *scalar HR_never=$Never/100
                         scalar TR=HR_CACE^(-1/W_shape)

                         postfile `sim' ITT ITT_l ITT_u psi psi_l psi_u b_cox b_cox_l b_cox_u ///
                              b_Weib b_Weib_l b_Weib_u Weib_shp HR__psi HR__psi_l HR__psi_u ///
                                          HR__ITT HR__ITT_l HR__ITT_u  endstudy  using results, replace
              quietly {
                         scalar N_simul=1000
                         forvalues i = 1/1000 {
                         replace t_pot=(-ln(uniform())/W_lamda)^(1/W_shape) if complier==1 & R==0
                         replace t_pot=(-ln(uniform())/(HR_CACE*W_lamda))^(1/W_shape) if complier==1 & R==1

                         replace U0=(-ln(uniform())/(HR_never*W_lamda))^(1/W_shape) if complier==0
                         *replace U0=(-ln(uniform())/(HR_always*W_lamda))^(1/W_shape) if complier==2
                         replace U1=U0*TR if complier==0
                         *replace U1=U0*TR if complier==0|complier==2
                         replace t_pot=U0 if complier==0 & R==0
                         *replace t_pot=U1 if complier==2 & R==1

                         replace xoyrs=U1*uniform() if complier==0 & R==1
                         replace t_pot=(U1-xoyrs)/TR+xoyrs if complier==0 & R==1

                         *replace xoyrs=U0*uniform() if complier==2 & R==0
                         *replace t_pot=U1-xoyrs*TR+xoyrs if complier==2 & R==0
                         *same as below since U0*TR=U1
                         *replace t_pot=(U0-xoyrs)*TR+xoyrs if complier==2 & R==0
```

```
			centile t_pot, centile(6.11)
			replace endFU=r(c_1)
			scalar endstudy=r(c_1)
		replace MACE=t_pot<=endFU
		replace t=min(t_pot, endFU)

			stset t, fail(MACE)
**ITT: Cox model HR estimate (natural-log-scale)
			stcox R
			matrix b=e(b)
			 matrix V=e(V)
			scalar b=b[1,1]
			scalar sd=sqrt(V[1,1])
			scalar b_cox=b
			scalar b_cox_l=b-invnorm(0.975)*sd
			scalar b_cox_u=b+invnorm(0.975)*sd

**ITT: Weibull model HR estimate (natural-log-scale)
			streg R, dist(weib)
			**also estimates Shape parameter
			scalar Weib_shp=e(aux_p)
			matrix b=e(b)
			matrix V=e(V)
			scalar b=b[1,1]
			scalar sd=sqrt(V[1,1])
			scalar b_Weib=b
			scalar b_Weib_l=b-invnorm(0.975)*sd
			scalar b_Weib_u=b+invnorm(0.975)*sd

**Causal: time ratio (TR=exp(-psi) comparing exposed vs unexposed)
   **here psi=[-Ln(TR)], i.e, on natural log scale, recensoring applied
			strbee R, xo1( xoyrs xo)  xo0(xoyrs xo) endstudy(endFU)
			scalar psi=r(psi)
			scalar psi_l=r(psilow)
			scalar psi_u=r(psiupp)

			**convert TR to HR (natural-log-scale)
			*Weibull model stimated shape parameter above Weib_shp, not pre-specified parameter W_shape
			**note: HR=TR^(-W_shape)=exp(-psi)^(-W_shape),
```

```
   **note:  ln(HR)=-W_shape*ln(TR)=(-W_shape)*(-psi)=W_shape*psi

		scalar HR__psi=psi*Weib_shp
		scalar HR__psi_l=psi_l*Weib_shp
		scalar HR__psi_u=psi_u*Weib_shp


**ITT estimates of TR(natural log scale) by Accelerate failure time model
strbee R
scalar ITT=r(psi)
scalar ITT_l=r(psilow)
scalar ITT_u=r(psiupp)
		scalar HR__ITT=ITT*Weib_shp
		scalar HR__ITT_l=ITT_l*Weib_shp
		scalar HR__ITT_u=ITT_u*Weib_shp

post `sim' (ITT) (ITT_l) (ITT_u) (psi) (psi_l) (psi_u) (b_cox) (b_cox_l) (b_cox_u)  ///
					(b_Weib) (b_Weib_l) (b_Weib_u) (Weib_shp) (HR__psi) (HR__psi_l) (HR__psi_u) ///
					 (HR__ITT) (HR__ITT_l) (HR__ITT_u)  (endstudy)

 *closes simulation loop of N_simul runs
 }
 *closes quietly
 }
 postclose `sim'

 ***Step 3b: summarize results of the N_simul simulations under each 1 parameter setting
use results, clear
quietly {
		foreach b in  ITT ITT_l ITT_u psi psi_l psi_u b_cox b_cox_l b_cox_u ///
					b_Weib b_Weib_l b_Weib_u Weib_shp HR__psi HR__psi_l HR__psi_u ///
					 HR__ITT HR__ITT_l HR__ITT_u  endstudy {
							sum `b', d
							scalar `b'_p25=r(p25)
							scalar `b'_p50=r(p50)
							scalar `b'_p75=r(p75)
							scalar `b'_avg=r(mean)
							scalar `b'_sd= r(sd)
							scalar `b'_min= r(min)
							scalar `b'_max= r(max)
```

```
}

count if b_cox_l<0 & b_cox_u<ln(1.3) & HR__psi_l<0 & HR__psi_u<ln(1.3)
scalar LULU0000=r(N)/N_simul
count if b_cox_l<0 & b_cox_u<ln(1.3) & HR__psi_l<0 & HR__psi_u>=ln(1.3)
scalar LULU0001=r(N)/N_simul
count if b_cox_l<0 & b_cox_u<ln(1.3) & HR__psi_l>=0 & HR__psi_u<ln(1.3)
scalar LULU0010=r(N)/N_simul
count if b_cox_l<0 & b_cox_u<ln(1.3) & HR__psi_l>=0 & HR__psi_u>=ln(1.3)
scalar LULU0011=r(N)/N_simul

count if b_cox_l<0 & b_cox_u>=ln(1.3) & HR__psi_l<0 & HR__psi_u<ln(1.3)
scalar LULU0100=r(N)/N_simul
count if b_cox_l<0 & b_cox_u>=ln(1.3) & HR__psi_l<0 & HR__psi_u>=ln(1.3)
scalar LULU0101=r(N)/N_simul
count if b_cox_l<0 & b_cox_u>=ln(1.3) & HR__psi_l>=0 & HR__psi_u<ln(1.3)
scalar LULU0110=r(N)/N_simul
count if b_cox_l<0 & b_cox_u>=ln(1.3) & HR__psi_l>=0 & HR__psi_u>=ln(1.3)
scalar LULU0111=r(N)/N_simul

count if b_cox_l>=0 & b_cox_u<ln(1.3) & HR__psi_l<0 & HR__psi_u<ln(1.3)
scalar LULU1000=r(N)/N_simul
count if b_cox_l>=0 & b_cox_u<ln(1.3) & HR__psi_l<0 & HR__psi_u>=ln(1.3)
scalar LULU1001=r(N)/N_simul
count if b_cox_l>=0 & b_cox_u<ln(1.3) & HR__psi_l>=0 & HR__psi_u<ln(1.3)
scalar LULU1010=r(N)/N_simul
count if b_cox_l>=0 & b_cox_u<ln(1.3) & HR__psi_l>=0 & HR__psi_u>=ln(1.3)
scalar LULU1011=r(N)/N_simul

count if b_cox_l>=0 & b_cox_u>=ln(1.3) & HR__psi_l<0 & HR__psi_u<ln(1.3)
scalar LULU1100=r(N)/N_simul
count if b_cox_l>=0 & b_cox_u>=ln(1.3) & HR__psi_l<0 & HR__psi_u>=ln(1.3)
scalar LULU1101=r(N)/N_simul
count if b_cox_l>=0 & b_cox_u>=ln(1.3) & HR__psi_l>=0 & HR__psi_u<ln(1.3)
scalar LULU1110=r(N)/N_simul
count if b_cox_l>=0 & b_cox_u>=ln(1.3) & HR__psi_l>=0 & HR__psi_u>=ln(1.3)
scalar LULU1111=r(N)/N_simul


****step 3c:save summary of N_simul simulations into 1 temporary dataset for appending
```

```
clear
set obs 1
foreach vn in  N N_simul p_trt  p_comply   W_shape W_lamda  seed ///
       HR_CACE HR_never  TR    ///
                         LULU0000 LULU0001 LULU0010  LULU0011 LULU0100 LULU0101 LULU0110  LULU0111 ///
           LULU1000 LULU1001 LULU1010 LULU1011 LULU1100 LULU1101 LULU1110 LULU1111 {
                        gen `vn'=`vn'
                                              }


foreach sn in  ITT ITT_l ITT_u psi psi_l psi_u b_cox b_cox_l b_cox_u ///
           b_Weib b_Weib_l b_Weib_u Weib_shp HR__psi HR__psi_l HR__psi_u ///
                                     HR__ITT HR__ITT_l HR__ITT_u endstudy  {

                                              gen `sn'_p25=`sn'_p25
                                              gen `sn'_p50=`sn'_p50
                                              gen `sn'_p75=`sn'_p75
                                              gen `sn'_avg=`sn'_avg
                                              gen `sn'_sd=`sn'_sd
                                              gen `sn'_min= `sn'_min
                                              gen `sn'_max= `sn'_max

                                                                                    }

             save temp_drop, replace
*close quietly
             }

use scafold, clear
append using temp_drop
save scafold, replace

*close Never loop
global Never=$Never+50
}
*close CACE loop

global CACE=$CACE+5
}
```

```
display "ended  on " c(current_date) " at "   c(current_time)

************************************************************************
***Step4: organize results for Excel file************************************
use scafold, clear

drop if HR_CACE==float(99.99)

save bkup_scafold, replace
save expo_always, replace

***Time ratio(TR) exponential different from HR: strbee parameter=exp(-psi) comparing exposed to unexposed

quietly {

foreach b in ITT ITT_l ITT_u psi psi_l psi_u {
replace `b'_p25=exp(-`b'_p25)
 replace `b'_p50=exp(-`b'_p50)
 replace `b'_p75=exp(-`b'_p75)
 replace `b'_avg=exp(-`b'_avg)
 replace `b'_sd=exp(-`b'_sd)
 replace `b'_min=exp(-`b'_min)
 replace `b'_max=exp(-`b'_max)
}

****Weib_shp should NOT be exponentiated, unlike other coefficent estimates

foreach b in  b_cox b_cox_l b_cox_u b_Weib b_Weib_l b_Weib_u  ///
		HR__psi HR__psi_l HR__psi_u HR__ITT HR__ITT_l HR__ITT_u {
replace `b'_p25=exp(`b'_p25)
 replace `b'_p50=exp(`b'_p50)
 replace `b'_p75=exp(`b'_p75)
 replace `b'_avg=exp(`b'_avg)
 replace `b'_sd=exp(`b'_sd)
 replace `b'_min=exp(`b'_min)

 replace `b'_max=exp(`b'_max)

 }
```

```
**close quietly

}

* format HR* %4.2f

* format b* %4.2f

save temp_drop, replace
gen UU00=LULU0000+LULU1010+LULU0010+LULU1000
gen UU01=LULU0001+LULU1001+LULU0011+LULU1011
gen UU10=LULU0100+LULU0110+LULU1100+LULU1110
gen UU11=LULU1111+LULU0101+LULU0111+LULU1101
label var UU00 "95%UB: ITT, causal both<1.3"
label var UU01 "95% UB:ITT<1.3, causal >=1.3"
label var UU11 "95%UB: ITT and causal both>=1.3"
label var UU10 "95%UB: ITT>=1.3 causal<1.3"

gen L0UU00=LULU0000+LULU0010
gen L0UU01=LULU0001+LULU0011
gen L0UU10=LULU0100+LULU0110
gen L0UU11=LULU0101+LULU0111

label var L0UU00 "95%UB:ITT,causal both<1.3; ITT LB<1"
label var L0UU01 "95% UB:ITT<1.3, causal >=1.3; ITT LB<1"
label var L0UU11 "95%UB: ITT and causal both>=1.3; ITT LB<1"
label var L0UU10 "95%UB: ITT>=1.3 causal<1.3; ITT LB<1"

gen L1UU00=UU00-L0UU00
gen L1UU01=UU01-L0UU01
gen L1UU10=UU10-L0UU10
gen L1UU11=UU11-L0UU11

label var L1UU00 "95%UB:ITT,causal both<1.3; ITT LB>=1"
label var L1UU01 "95% UB:ITT<1.3, causal >=1.3; ITT LB>=1"
label var L1UU11 "95%UB: ITT and causal both>=1.3; ITT LB>=1"
label var L1UU10 "95%UB: ITT>=1.3 causal<1.3; ITT LB>=1"
*gen L0=LULU0000+LULU0001+LULU0011+LULU0010+LULU0100+LULU0101+LULU0110+LULU0111
*gen cL0UU00=(LULU0000+LULU0010)/L0
*gen cL0UU01=(LULU0001+LULU0011)/L0
```

```
*gen cL0UU10=(LULU0100+LULU0110)/L0
*gen cL0UU11=(LULU0101+LULU0111)/L0
*label var L0UU00 "95%UB:ITT,causal both<1.3|ITT LB<1"
*label var L0UU01 "95% UB:ITT<1.3, causal >=1.3|ITT LB<1"
*label var L0UU11 "95%UB: ITT and causal both>=1.3|ITT LB<1"
*label var L0UU10 "95%UB: ITT>=1.3 causal<1.3|ITT LB<1"
drop if HR_CACE==float(99.99)

save temp_drop, replace

format seed %10.0f

order HR_CACE HR_never p_comply UU11 UU10 UU01 UU00 L0UU11 L0UU01 L0UU00 L1UU11 L1UU01  L1UU00, first
bro

reshape long b_cox b_Weib HR__psi HR__ITT  ITT psi ///
       b_cox_l b_Weib_l HR__psi_l HR__ITT_l ITT_l psi_l ///
                     b_cox_u b_Weib_u HR__psi_u HR__ITT_u ITT_u psi_u Weib_shp endstudy, i(HR_CACE HR_never) j(param) string
sort HR_CACE HR_never  b_cox
format seed %10.0f

order HR_CACE HR_never  param b_cox HR__psi b_cox_u HR__psi_u b_cox_l HR__psi_l, first
bro

**Note: for parameters with distribution estimated percentile, range, mean, sd,
**Parameter estimates starting with ITT_: come from xtrbee on Randomized trt, ignoring compliance, time ratio
**Parameter estimates starting with psi_: come from xtrbee considering compliance, time ratio
**Parameter estimates starting with b_cox: come from Cox regression on Randomized trt, Hazard ratio
**estimates starting with b_Weib: parametric Weibull regression ITT analysis, Hazard ratio
**Weib_shp: parametric Weibull regression ITT analysis, shape parameter for Weibull survival distribution
**HR__psi: hazard ratio converted from causal time ratio (xtrbee considering compliance) and Weibull shape param
**HR__ITT: hazard ratio converted from time ratio (xtrbee ITT ignoring compliance) and Weibull shape parameter
**check that HR__ITT: and b_cox:  and b_Weib: to be (almost) identical
*******end of program*******
```

Module 3: Cross-over(always-taker), premature discontinuation (never-taker)

```
clear all
set more off
cd \UserDefinedDirectory
```

```
**note: HR=TR^(-W_shape), log(HR)=-W_shape*log(TR)
*******************step 1************************
*generate a scaffold dataset for appending N_simul rounds of results
set obs 1
foreach x in  N N_simul p_trt  p_comply p_never p_always W_shape W_lamda  ///
        HR_CACE HR_never HR_always TR  seed    {
                                                   gen `x'=99.99
                                                                                     }
                label var N "==Total sample size, randomized, treatment+ctrl"
                label var N_simul "#simulations for each unique parameter combo"
                label var p_trt "==% assigned to treatment arm"
                label var p_comply "==% compliers, same across 2 arms by randomization"
                label var p_never "==% never-taker, same across 2 arms by randomization"
                label var p_always "==%always-taker=1-%compliers-%never-takers"
                label var W_shape "==Weibull shape param,based on ADVANCE+Framingham, fixed"
                label var W_lamda "==Weibull scale param, unexposed complier"

                label var HR_CACE "==HR:trt effect in compliers"
                label var HR_never "==HR: never-users vs unexposed compliers"
                label var HR_always "==HR: always-takers vs unexposed compliers"
                label var TR "==Causal survival time ratio: exposed vs unexposed compliers"
                label var seed "Random seed"


foreach y in LULU0000 LULU0001 LULU0010  LULU0011 LULU0100 LULU0101 LULU0110  LULU0111 ///
        LULU1000 LULU1001 LULU1010 LULU1011 LULU1100 LULU1101 LULU1110 LULU1111 {
          gen `y'=0
                                              }
                label var LULU0000 "ITT lower bound<1, upper bound<1.3, causal LB<1, UB<1.3"
                label var LULU1111 "ITT 95%LB>=1, UB>=1.3, causal LB>=1, UB>=1.3"


foreach b in  ITT ITT_l ITT_u psi psi_l psi_u b_cox b_cox_l b_cox_u ///
        b_Weib b_Weib_l b_Weib_u Weib_shp HR__psi HR__psi_l HR__psi_u ///
                                 HR__ITT HR__ITT_l HR__ITT_u  endstudy {
 gen `b'_p25=99.99
 gen `b'_p50=99.99
 gen `b'_p75=99.99
 gen `b'_avg=99.99
 gen `b'_sd=99.99
 gen `b'_min=99.99
 gen `b'_max=99.99

 label var `b'_p25 "25th pctile, `b'"
 label var `b'_p50 "median, `b'"
 label var `b'_p75 "75th pctile, `b'"

 label var `b'_sd "SD, `b'"
 label var `b'_min "min, `b'"
 label var `b'_max "max, `b'"
 }
```

```
label var ITT_avg "mean, point est ITT-TR(rbee ignoring xo)"
label var ITT_l_avg "mean, LCL of ITT-TR"
label var ITT_u_avg "mean, UCL of ITT-TR"
label var psi_avg "mean, point est psi-TR(rbee handling xo)"
label var psi_l_avg "mean, LCL of psi-TR"
label var b_cox_avg "mean, point est ITT-Cox-HR"
label var b_Weib_avg "mean, point est ITT-Weibull HR"
label var HR__psi_avg "mean, point est psi-HR(psi-TR^(-Weib_shp))"
label var HR__ITT_avg "mean, point est ITT-HR(ITT-TR^(-Weib_shp"
save scafold, replace
clear all

********************step 2************************
*set up data frame for running each 1 simulation
quietly {
	set obs 3
	gen complier=_n-1
	expand 2
	*label define complier 0 "Never-taker" 1 "Complier" 2 "Always-taker"
	gen R=_n>3
	***sample size=N
	scalar N=10000
	***in theory, all N subjects will have MACE event when FU through largest potential life-time t_pot
	***percentile of t_pot to end FU/admin censor when 611 MACE events occur (90%power to exclude UB 1.3)
	scalar EvntDriven=611*100/N
	**allocation ratio
	scalar p_trt=0.5
	scalar p_comply=0.8
	scalar p_never=0.1
*can change parameter settings: p_comply=0.6  and p_never=0.2, for example
	scalar p_always=1-p_comply-p_never


	gen expan=N*p_trt*p_comply if complier==1 &  R==1
	replace expan=N*(1-p_trt)*p_comply if complier==1 &  R==0
	replace expan=N*p_trt*p_never   if complier==0 &  R==1
	replace expan=N*(1-p_trt)*p_never   if complier==0 &  R==0
	replace expan=N*p_trt*p_always   if complier==2 &  R==1
	replace expan=N*(1-p_trt)*p_always   if complier==2 &  R==0
	expand expan

	*table R complier, row col c(freq sum D)
	***generate subject id for future x-over studies
	gen id=_n


	gen xo=1 if complier==0 & R==1
	replace xo=1 if complier==2 & R==0
	foreach x in t_pot U0 U1 xoyrs t MACE  endFU {
		gen `x'=.
								}
```

```
                scalar seed=822246
                set seed 822246

save tempframe, replace
*closes quietly loop
}

********step 3: run simulation for N_simul times, each time using tempframe.dta*******
 ***Step 3a: simulation runs N_simul times on data frame
 nois _dots 0, title(Loop running) reps(5)
tempname sim
scalar W_shape=1.223548
scalar W_lamda=0.02470739

global CACE = 130
display "program begins on " c(current_date) " at "   c(current_time)
while $CACE<=130  {

global Never=50
   while $Never<=200 {

                nois _dots `i' 0
   use tempframe, clear
                                scalar HR_CACE=$CACE/100
                                *scalar HR_always=$Never/100
                                *scalar HR_never=100/$Never
                                scalar HR_never=$Never/100
                                scalar HR_always=0.5

                                *scalar HR_always=100/$Never
                                *scalar HR_never=$Never/100
                                scalar TR=HR_CACE^(-1/W_shape)

                                postfile `sim' ITT ITT_l ITT_u psi psi_l psi_u b_cox b_cox_l b_cox_u ///
                                     b_Weib b_Weib_l b_Weib_u Weib_shp HR__psi HR__psi_l HR__psi_u ///
                                              HR__ITT HR__ITT_l HR__ITT_u  endstudy  using results, replace
                quietly {
                                scalar N_simul=1000
                                forvalues i = 1/1000 {
                                replace t_pot=(-ln(uniform())/W_lamda)^(1/W_shape) if complier==1 & R==0
                                replace t_pot=(-ln(uniform())/(HR_CACE*W_lamda))^(1/W_shape) if complier==1 & R==1

                                replace U0=(-ln(uniform())/(HR_never*W_lamda))^(1/W_shape) if complier==0
                                replace U0=(-ln(uniform())/(HR_always*W_lamda))^(1/W_shape) if complier==2

                                replace U1=U0*TR if complier==0|complier==2
                                replace t_pot=U0 if complier==0 & R==0
                                replace t_pot=U1 if complier==2 & R==1

                                replace xoyrs=U1*uniform() if complier==0 & R==1
                                replace t_pot=(U1-xoyrs)/TR+xoyrs if complier==0 & R==1
```

```
replace xoyrs=U0*uniform() if complier==2 & R==0
replace t_pot=U1-xoyrs*TR+xoyrs if complier==2 & R==0
*same as below since U0*TR=U1
*replace t_pot=(U0-xoyrs)*TR+xoyrs if complier==2 & R==0

centile t_pot, centile(6.11)
replace endFU=r(c_1)
scalar endstudy=r(c_1)
replace MACE=t_pot<=endFU
replace t=min(t_pot, endFU)

stset t, fail(MACE)
**ITT: Cox model HR estimate (natural-log-scale)
stcox R
matrix b=e(b)
 matrix V=e(V)
scalar b=b[1,1]
scalar sd=sqrt(V[1,1])
scalar b_cox=b
scalar b_cox_l=b-invnorm(0.975)*sd
scalar b_cox_u=b+invnorm(0.975)*sd

**ITT: Weibull model HR estimate (natural-log-scale)
streg R, dist(weib)
**also estimates Shape parameter
scalar Weib_shp=e(aux_p)
matrix b=e(b)
matrix V=e(V)
scalar b=b[1,1]
scalar sd=sqrt(V[1,1])
scalar b_Weib=b
scalar b_Weib_l=b-invnorm(0.975)*sd
scalar b_Weib_u=b+invnorm(0.975)*sd

**Causal: time ratio (TR=exp(-psi) comparing exposed vs unexposed)
   **here psi=[-Ln(TR)], i.e, on natural log scale, recensoring applied
strbee R, xo1( xoyrs xo)  xo0(xoyrs xo) endstudy(endFU)
scalar psi=r(psi)
scalar psi_l=r(psilow)
scalar psi_u=r(psiupp)

**convert TR to HR (natural-log-scale)
*Weibull model stimated shape parameter above Weib_shp, not pre-specified parameter W_shape
**note: HR=TR^(-W_shape)=exp(-psi)^(-W_shape),
**note:  ln(HR)=-W_shape*ln(TR)=(-W_shape)*(-psi)=W_shape*psi

scalar HR__psi=psi*Weib_shp
scalar HR__psi_l=psi_l*Weib_shp
scalar HR__psi_u=psi_u*Weib_shp
```

```
**ITT estimates of TR(natural log scale) by Accelerate failure time model
strbee R
scalar ITT=r(psi)
scalar ITT_l=r(psilow)
scalar ITT_u=r(psiupp)
        scalar HR__ITT=ITT*Weib_shp
        scalar HR__ITT_l=ITT_l*Weib_shp
        scalar HR__ITT_u=ITT_u*Weib_shp

post `sim' (ITT) (ITT_l) (ITT_u) (psi) (psi_l) (psi_u) (b_cox) (b_cox_l) (b_cox_u)  ///
                    (b_Weib) (b_Weib_l) (b_Weib_u) (Weib_shp) (HR__psi) (HR__psi_l) (HR__psi_u) ///
                     (HR__ITT) (HR__ITT_l) (HR__ITT_u)  (endstudy)

 *closes simulation loop of N_simul runs
 }
 *closes quietly
 }
 postclose `sim'

 ***Step 3b: summarize results of the N_simul simulations under each 1 parameter setting
use results, clear
quietly {
        foreach b in  ITT ITT_l ITT_u psi psi_l psi_u b_cox b_cox_l b_cox_u ///
                    b_Weib b_Weib_l b_Weib_u Weib_shp HR__psi HR__psi_l HR__psi_u ///
                     HR__ITT HR__ITT_l HR__ITT_u  endstudy {
                                sum `b', d
                                scalar `b'_p25=r(p25)
                                scalar `b'_p50=r(p50)
                                scalar `b'_p75=r(p75)
                                scalar `b'_avg=r(mean)
                                scalar `b'_sd= r(sd)
                                scalar `b'_min= r(min)
                                scalar `b'_max= r(max)
                                                                        }

count if b_cox_l<0 & b_cox_u<ln(1.3) & HR__psi_l<0 & HR__psi_u<ln(1.3)
scalar LULU0000=r(N)/N_simul
count if b_cox_l<0 & b_cox_u<ln(1.3) & HR__psi_l<0 & HR__psi_u>=ln(1.3)
scalar LULU0001=r(N)/N_simul
count if b_cox_l<0 & b_cox_u<ln(1.3) & HR__psi_l>=0 & HR__psi_u<ln(1.3)
scalar LULU0010=r(N)/N_simul
count if b_cox_l<0 & b_cox_u<ln(1.3) & HR__psi_l>=0 & HR__psi_u>=ln(1.3)
scalar LULU0011=r(N)/N_simul

count if b_cox_l<0 & b_cox_u>=ln(1.3) & HR__psi_l<0 & HR__psi_u<ln(1.3)
scalar LULU0100=r(N)/N_simul
count if b_cox_l<0 & b_cox_u>=ln(1.3) & HR__psi_l<0 & HR__psi_u>=ln(1.3)
scalar LULU0101=r(N)/N_simul
count if b_cox_l<0 & b_cox_u>=ln(1.3) & HR__psi_l>=0 & HR__psi_u<ln(1.3)
scalar LULU0110=r(N)/N_simul
count if b_cox_l<0 & b_cox_u>=ln(1.3) & HR__psi_l>=0 & HR__psi_u>=ln(1.3)
scalar LULU0111=r(N)/N_simul
```

```
		count if b_cox_l>=0 & b_cox_u<ln(1.3) & HR__psi_l<0 & HR__psi_u<ln(1.3)
		scalar LULU1000=r(N)/N_simul
		count if b_cox_l>=0 & b_cox_u<ln(1.3) & HR__psi_l<0 & HR__psi_u>=ln(1.3)
		scalar LULU1001=r(N)/N_simul
		count if b_cox_l>=0 & b_cox_u<ln(1.3) & HR__psi_l>=0 & HR__psi_u<ln(1.3)
		scalar LULU1010=r(N)/N_simul
		count if b_cox_l>=0 & b_cox_u<ln(1.3) & HR__psi_l>=0 & HR__psi_u>=ln(1.3)
		scalar LULU1011=r(N)/N_simul

		count if b_cox_l>=0 & b_cox_u>=ln(1.3) & HR__psi_l<0 & HR__psi_u<ln(1.3)
		scalar LULU1100=r(N)/N_simul
		count if b_cox_l>=0 & b_cox_u>=ln(1.3) & HR__psi_l<0 & HR__psi_u>=ln(1.3)
		scalar LULU1101=r(N)/N_simul
		count if b_cox_l>=0 & b_cox_u>=ln(1.3) & HR__psi_l>=0 & HR__psi_u<ln(1.3)
		scalar LULU1110=r(N)/N_simul
		count if b_cox_l>=0 & b_cox_u>=ln(1.3) & HR__psi_l>=0 & HR__psi_u>=ln(1.3)
		scalar LULU1111=r(N)/N_simul



****step 3c:save summary of N_simul simulations into 1 temporary dataset for appending

clear
set obs 1
foreach vn in  N N_simul p_trt  p_comply p_never p_always W_shape W_lamda  seed ///
	HR_CACE HR_never HR_always TR    ///
				LULU0000 LULU0001 LULU0010  LULU0011 LULU0100 LULU0101 LULU0110  LULU0111 ///
		LULU1000 LULU1001 LULU1010 LULU1011 LULU1100 LULU1101 LULU1110 LULU1111 {
			gen `vn'=`vn'
					}


foreach sn in  ITT ITT_l ITT_u psi psi_l psi_u b_cox b_cox_l b_cox_u ///
		b_Weib b_Weib_l b_Weib_u Weib_shp HR__psi HR__psi_l HR__psi_u ///
					HR__ITT HR__ITT_l HR__ITT_u endstudy  {

						gen `sn'_p25=`sn'_p25
						gen `sn'_p50=`sn'_p50
						gen `sn'_p75=`sn'_p75
						gen `sn'_avg=`sn'_avg
						gen `sn'_sd=`sn'_sd
						gen `sn'_min= `sn'_min
						gen `sn'_max= `sn'_max

												}

		save temp_drop, replace
*close quietly
		}

use scafold, clear
```

```
append using temp_drop
save scafold, replace

*close Never loop
global Never=$Never+50
}
*close CACE loop

global CACE=$CACE+10
}
display "ended  on " c(current_date) " at "   c(current_time)

****************************************************************************
***Step4: organize results for Excel file************************************
use scafold, clear
***copy and paste to Excel****


use scafold, clear

drop if HR_CACE==float(99.99)

save bkup_scafold, replace
save expo_always, replace

***Time ratio(TR) exponential different from HR: strbee parameter=exp(-psi) comparing exposed to unexposed

quietly {

foreach b in ITT ITT_l ITT_u psi psi_l psi_u {
replace `b'_p25=exp(-`b'_p25)
 replace `b'_p50=exp(-`b'_p50)
 replace `b'_p75=exp(-`b'_p75)
 replace `b'_avg=exp(-`b'_avg)
 replace `b'_sd=exp(-`b'_sd)
 replace `b'_min=exp(-`b'_min)
 replace `b'_max=exp(-`b'_max)
}

****Weib_shp should NOT be exponentiated, unlike other coefficent estimates

foreach b in  b_cox b_cox_l b_cox_u b_Weib b_Weib_l b_Weib_u  ///
               HR__psi HR__psi_l HR__psi_u HR__ITT HR__ITT_l HR__ITT_u {
replace `b'_p25=exp(`b'_p25)
 replace `b'_p50=exp(`b'_p50)
 replace `b'_p75=exp(`b'_p75)
 replace `b'_avg=exp(`b'_avg)
 replace `b'_sd=exp(`b'_sd)
 replace `b'_min=exp(`b'_min)
 replace `b'_max=exp(`b'_max)

}
```

```
**close quietly
}
save expo_always, replace


use expo_always, clear

* format HR* %4.2f
* format b* %4.2f

save temp_drop, replace

gen UU00=LULU0000+LULU1010+LULU0010+LULU1000
gen UU01=LULU0001+LULU1001+LULU0011+LULU1011
gen UU10=LULU0100+LULU0110+LULU1100+LULU1110
gen UU11=LULU1111+LULU0101+LULU0111+LULU1101
label var UU00 "95%UB: ITT, causal both<1.3"
label var UU01 "95% UB:ITT<1.3, causal >=1.3"
label var UU11 "95%UB: ITT and causal both>=1.3"
label var UU10 "95%UB: ITT>=1.3 causal<1.3"

gen L0UU00=LULU0000+LULU0010
gen L0UU01=LULU0001+LULU0011
gen L0UU10=LULU0100+LULU0110
gen L0UU11=LULU0101+LULU0111

label var L0UU00 "95%UB:ITT,causal both<1.3; ITT LB<1"
label var L0UU01 "95% UB:ITT<1.3, causal >=1.3; ITT LB<1"
label var L0UU11 "95%UB: ITT and causal both>=1.3; ITT LB<1"
label var L0UU10 "95%UB: ITT>=1.3 causal<1.3; ITT LB<1"

gen L1UU00=UU00-L0UU00
gen L1UU01=UU01-L0UU01
gen L1UU10=UU10-L0UU10
gen L1UU11=UU11-L0UU11

label var L1UU00 "95%UB:ITT,causal both<1.3; ITT LB>=1"
label var L1UU01 "95% UB:ITT<1.3, causal >=1.3; ITT LB>=1"
label var L1UU11 "95%UB: ITT and causal both>=1.3; ITT LB>=1"
label var L1UU10 "95%UB: ITT>=1.3 causal<1.3; ITT LB>=1"
*gen L0=LULU0000+LULU0001+LULU0011+LULU0010+LULU0100+LULU0101+LULU0110+LULU0111
*gen cL0UU00=(LULU0000+LULU0010)/L0
*gen cL0UU01=(LULU0001+LULU0011)/L0
*gen cL0UU10=(LULU0100+LULU0110)/L0
*gen cL0UU11=(LULU0101+LULU0111)/L0
*label var L0UU00 "95%UB:ITT,causal both<1.3|ITT LB<1"
*label var L0UU01 "95% UB:ITT<1.3, causal >=1.3|ITT LB<1"
*label var L0UU11 "95%UB: ITT and causal both>=1.3|ITT LB<1"
*label var L0UU10 "95%UB: ITT>=1.3 causal<1.3|ITT LB<1"
save temp_drop, replace

format seed %10.0f
```

```
*order HR_CACE HR_never HR_always p_comply p_never UU11 UU10 UU01 UU00 L0UU11 L0UU01 L0UU00 L1UU11 L1UU01  L1UU00, first
order HR_CACE HR_never HR_always p_comply p_never UU11 UU10 UU01 UU00 L0UU11 L0UU01 L0UU00 L1UU11 L1UU01  L1UU00, first


bro

*describe, replace
*bro name varlab
save temp_drop, replace

use temp_drop, clear
bro
/*


reshape long b_cox b_Weib HR__psi HR__ITT  ITT psi ///
        b_cox_l b_Weib_l HR__psi_l HR__ITT_l ITT_l psi_l ///
                                b_cox_u b_Weib_u HR__psi_u HR__ITT_u ITT_u psi_u Weib_shp endstudy, i(HR_CACE HR_never HR_always) j(param) string
sort HR_CACE HR_never HR_always b_cox
format seed %10.0f

order HR_CACE HR_never HR_always p_comply p_never param b_cox HR__psi b_cox_u HR__psi_u b_cox_l HR__psi_l, first
bro

**Note: for parameters with distribution estimated percentile, range, mean, sd,
**Parameter estimates starting with ITT_: come from xtrbee on Randomized trt, ignoring compliance, time ratio
**Parameter estimates starting with psi_: come from xtrbee considering compliance, time ratio
**Parameter estimates starting with b_cox: come from Cox regression on Randomized trt, Hazard ratio
**estimates starting with b_Weib: parametric Weibull regression ITT analysis, Hazard ratio
**Weib_shp: parametric Weibull regression ITT analysis, shape parameter for Weibull survival distribution
**HR__psi: hazard ratio converted from causal time ratio (xtrbee considering compliance) and Weibull shape param
**HR__ITT: hazard ratio converted from time ratio (xtrbee ITT ignoring compliance) and Weibull shape parameter
**check HR__ITT: and b_cox:  and b_Weib: to be (almost) identical
*******end of program*******
```
